# pH-responsive nano-vaccine combined with anti-PD-1 antibodies for enhanced immunotherapy of breast cancer

**DOI:** 10.7150/thno.107200

**Published:** 2025-04-28

**Authors:** Ning Wang, Hong Yu, Jianqiao Yin, Xiaopeng Yu

**Affiliations:** 1 Department of Surgery, Shengjing Hospital of China Medical University, Shenyang 110004, China.; 2 Department of Oncology, Shengjing Hospital of China Medical University, Shenyang 110004, China.

**Keywords:** nano-vaccine, programmed cell death protein 1, breast cancer, immunotherapy, tumor microenvironment, antigen delivery, immune memory, tumor metastasis

## Abstract

**Objective:** This study aimed to investigate the therapeutic potential and underlying mechanisms of a novel pH-responsive nano-vaccine in combination with anti-Programmed Cell Death Protein 1 (PD-1) antibodies for the treatment of breast cancer (BC), with a focus on tumor growth inhibition, metastasis prevention, and immune microenvironment modulation.

**Methods:** A pH-responsive amphiphilic diblock copolymer was synthesized using reversible addition-fragmentation chain transfer (RAFT) polymerization and conjugated with STING agonist ADU-S100 and mannose to specifically target dendritic cells (DCs). The nano-vaccine was further formulated with antigen peptides and polyethyleneimine (PEI) to enhance antigen delivery. Its particle size, stability, and surface charge were characterized using dynamic light scattering (DLS) and zeta potential analysis. *In vitro*, the immunostimulatory capacity of the nano-vaccine was evaluated via flow cytometry (FCM) analysis of DC activation markers. *In vivo*, mouse immune and tumor recurrence models were used to assess the its effects on T-cell activation, tumor suppression, and immune memory induction. The therapeutic efficacy of nano-vaccine/anti-PD-1 combination therapy was further assessed.

**Results:** The nano-vaccine efficiently activated DCs and promoted antigen presentation, as indicated by increased CD80, CD86, and MHC-II expression *in vitro*. In mouse models, it effectively inhibited tumor growth, induced antigen-specific T-cell responses, and suppressed recurrent and metastatic tumor progression. The combination with anti-PD-1 antibodies further enhanced tumor control, immune cell infiltration, and survival rates compared to monotherapy.

**Conclusion:** The pH-responsive nano-vaccine combined with anti-PD-1 antibodies showed remarkable synergistic effects in BC treatment, highlighting its potential to enhance immune checkpoint blockade therapy and offer a promising strategy for clinical applications in solid tumors.

## Introduction

Breast cancer (BC), one of the most common malignant tumors among women worldwide, continues to see rising incidence rates, posing a significant threat to women's health 1-3. According to the World Health Organization, BC has the highest incidence and mortality rates among women's cancers, with millions diagnosed annually and a substantial number of fatalities 4-6. Despite significant advancements in BC treatment options, including surgery, radiotherapy, chemotherapy, and targeted therapies, which are highly effective in early-stage BC, challenges remain in the treatment of advanced or recurrent cases 7-9. This is particularly true for patients with metastatic BC, where the limitations of conventional therapies are pronounced, and outcomes often remain unsatisfactory 10, 11.

The tumor microenvironment in BC is complex and varied, comprising diverse immune cells, cytokines, and other signaling molecules. These components interact and impact tumor growth, metastasis, and therapeutic response 12-14. Within this environment, specific immune cells, such as tumor-associated macrophages (TAMs) and regulatory T-cells play crucial roles. They help cancer cells evade the body's immune surveillance by secreting immunosuppressive factors or expressing inhibitory molecules 15, 16, 12. The Programmed Cell Death Protein 1 (PD-1)/Programmed Death-Ligand 1 (PD-L1) pathway is one of the most extensively investigated immune checkpoints, where PD-L1 expressed on tumor cells or within the tumor microenvironment binds to PD-1 on immune cells, leading to immune suppression and facilitating immune escape 17-19. Therefore, disrupting this immune evasion mechanism to restore the body's immune surveillance and clearance functions presents a significant challenge in BC treatment 20-22.

In recent years, advances in immunotherapy, particularly immune checkpoint inhibitors (ICIs), have brought new hope for the treatment of BC 23, 24. Anti-PD-1 and anti-PD-L1 antibodies have demonstrated effectiveness in various cancer treatments, including advanced melanoma and non-small cell lung cancer, yet their application in BC has been less effective 25-27. The distinctive immune environment of BC means that single-agent immune checkpoint inhibition often fails to achieve optimal outcomes, necessitating combination with other treatments such as chemotherapy and targeted therapy to enhance efficacy 28, 15, 29. Additionally, selecting the appropriate patient cohort to optimize treatment personalization and precision remains a key focus and challenge in current research 30-32.

Nanotechnology, with its unique physical and chemical properties, is increasingly utilized in cancer therapy 33-35. Nanoparticles are designed to deliver drugs, genes, or other therapeutic molecules directly to tumor tissues, minimizing damage to normal tissues 36. In this study, we developed a novel targeted nano-vaccine based on nanotechnology. To synthesize the nano-vaccine, an amphiphilic diblock copolymer PEG-b-PDPA was prepared using RAFT polymerization, utilizing water-soluble polyethylene glycol (PEG) with a chain transfer agent and diisopropylamino ethyl methacrylate (DPA) monomer with a double bond 37-39. Due to the presence of tertiary amine (-N(iPr)_2_) groups in DPA, protonation occurs under acidic conditions, leading to a hydrophobic-to-hydrophilic transition, thereby enabling pH-responsive drug release 40, 41, 37. ADU-S100 was further modified via acrylate functionalization, introducing unsaturated double bonds that facilitated its polymerization onto the PEG-b-PDPA backbone. To achieve targeted delivery of the nano-vaccine, dextran was chemically conjugated to the PEG hydrophilic segment at the chain end of PEG-b-PDPA 42, 43. This vaccine encapsulates a STING agonist and a new antigen, specifically activating dendritic cells (DCs), enhancing their antigen presentation efficiency, and eliciting a strong T-cell immune response. Such nanotechnology-based vaccines not only improve treatment specificity but also enhance immune responses by modulating the tumor microenvironment.

This research aims to explore the mechanisms and therapeutic potential of a novel pH-responsive nano-vaccine combined with anti-PD-1 antibodies in BC treatment. By leveraging this innovative combination therapy, we aim to effectively suppress tumor growth, metastasis, and recurrence while enhancing immune clearance of tumor cells. Furthermore, this strategy may also provide novel insights and approaches for the treatment of other solid tumors. From a scientific perspective, this study investigates the immunological mechanisms underlying the combined application of nano-vaccine and ICIs, providing a theoretical basis and experimental data for future clinical applications. From a clinical standpoint, this research aims to establish a new and effective treatment regimen for BC, improving patient survival rates and quality of life, offering significant clinical value and translational potential.

## Materials and Methods

### Chemicals and materials

Dichloromethane (DCM, anhydrous, 34856), 2-(7-Azabenzotriazol-1-yl)-N, N, N', N'-tetramethyluronium hexafluorophosphate (HATU, 10873), Triethylamine (TEA, T0886), hydroxyl benzotrizole (HOBT, 711489), N, N-Dimethylformamide (DMF, 227056), azodiisobutyronitrile (AIBN, 714887), 1-(3-Dimethylaminopropyl)-3-ethylcarbodiimide hydrochloride (EDCI, 341006), diisopropylamino ethyl methacrylate (DPA, 730971), phosphotungstic acid (PTA, 2% w/v, 496626), tetrahydrofuran (THF, 401757), dimethyl sulfoxide (DMSO, 34943) were all ordered from Sigma-Aldrich China. ADU-S100 (TMLT10252L3MG100) was ordered from Chinese Medicine Reagent, China. α-D-Mannopyranosylphenyl isothiocyanate (Dex, XW06046821), M32 (SHRSCSHQTSAPSPKALAHNGTPRNAI) were obtained from Beijing Chemsynlab Pharmaceutical Science & Technology Co., Ltd.

### Cell lines and animals

The 4T1 mouse mammary tumor cells (CRL-2539, ATCC) and 4T1-Luc (CRL-2539-LUC2, ATCC) were obtained from the ATCC cell bank. Bone marrow precursors were harvested from the femurs of BALB/c mice and washed with 10% FBS RPMI-1640 medium (R8758, Sigma). These cells were then cultured at a density of 2 × 10^5^ cells/mL in modified DMEM (11965092, Thermo, USA), supplemented with 10% fetal bovine serum (10099141C, Thermo, USA), 100 U/mL penicillin, and 100 µg/mL streptomycin (10378016, Thermo, USA), along with 1.5 mM L-glutamine (21051040, Thermo, USA). During the culture process, 100 ng/mL mouse G-CSF (Catalog #: 414-CS, R&D Systems), 250 U/mL mouse GM-CSF (Catalog #: 415-ML, R&D Systems), and 80 ng/mL IL-4 (Catalog #: 404-ML, R&D Systems) were added to generate bone marrow-derived DCs (BMDCs) 44.

Four-week-old BALB/c mice (18-20 g, female) were purchased from our institution's Experimental Animal Research Center. The animals were maintained in an environment with a temperature of 25 ± 2 °C, humidity of 50 ± 5%, and a 12 h light/dark cycle, with free access to standard food and water. Humane endpoints included tumor burden exceeding 10% of body weight, weight loss exceeding 20% of body weight, ulceration at the tumor growth site, and persistent self-harm. These humane endpoints were approved by the Certification and Accreditation Administration of the People's Republic of China (CNCA). Euthanasia was performed using cervical dislocation under deep anesthesia. All animal studies were conducted in accordance with the guidelines of Shengjing Hospital, China Medical University, for the care and use of laboratory animals (Approval No.: CMUXN2022121).

### Stepwise synthesis of pH-sensitive polymers

Naming of compounds and synthetic materials refers to Table S1. To fabricate the nano-vaccine, an amphiphilic diblock copolymer, PEG-b-PDPA, was synthesized via reversible addition-fragmentation chain transfer (RAFT) polymerization. ADU-S100 was grafted onto the main chain of PEG-b-PDPA through the addition reaction of its unsaturated double bonds. To enhance the nano-vaccine targeting, a dextrorotatory sugar was chemically conjugated to the hydrophilic PEG end of the PEG-b-PDPA.

In anhydrous N,N-dimethylformamide (DMF), 4-cyano-4-(dodecylthiocarbonothioylthio) pentanoic acid (CTA, 0.048 mmol), 1-(3-dimethylaminopropyl)-3-ethylcarbodiimide hydrochloride (EDCI, 0.144 mmol), and 1-hydroxybenzotriazole (HOBT, 0.144 mmol) were dissolved and stirred for 1.5 h. Subsequently, methyl-capped PEG-NH_2_ (0.040 mmol) and TEA (0.160 mmol) were added under ice-bath conditions and allowed to react for 24 h. Upon completion, unreacted reagents were removed via dialysis, and the product was freeze-dried to obtain mPEG_108_-CTA. Next, mPEG_108_-CTA (0.019 mmol) was used as a macro-RAFT agent, dissolved in anhydrous DMF along with 2-(diisopropylamino) ethyl methacrylate (DPA, 1.114 mmol), and 2,2'-azobisisobutyronitrile (AIBN, 0.0019 mmol). The solution underwent three freeze-thaw cycles to remove oxygen, followed by reaction at 70 °C for 24 h in an oil bath. The resulting product was purified via dialysis, freeze-dried, and characterized by ^1^H-NMR (Figure S1).

ADU-S100 was chemically modified with methacrylic anhydride (MAAH) under anhydrous and oxygen-free conditions to introduce methacryloyl groups. All glassware was dried at 120 °C for 2 h and maintained under a nitrogen (N_2_) atmosphere. ADU-S100 (0.1 mmol) was dissolved in anhydrous DMF (5 mL) and stirred in an ice bath (0 °C). Anhydrous TEA (1.2 eq, 0.12 mmol) was added as a base, followed by the slow addition of MAAH (1.1 eq, 0.11 mmol). After 10 min, the mixture was stirred for another 30 min, then warmed to 25 °C and further stirred for 6-12 h. Upon completion, cold EtOAc (10 mL) was added, and the mixture was left to stand for 10 min before washing with 5% NaHCO_3_ (5 mL × 2). The organic layer was dried over Na_2_SO₄ for 30 min, filtered, and concentrated under vacuum evaporation to yield a yellow solid or oil-like product. The final product was purified using silica gel chromatography (CH_2_Cl_2_/MeOH = 10:1) and obtained as a light yellow powder or transparent oil, confirmed by ^1^H-NMR (Figure S2).

In anhydrous DMF, mPEG_108_-CTA (0.019 mmol), DPA (1.114 mmol), and ADU-S100 methacrylate (0.296 mmol) were dissolved. AIBN (0.0019 mmol) was added, followed by three vacuum-nitrogen cycles to remove oxygen. The reaction was conducted at 70 °C for 24 h in an oil bath. After completion, unreacted monomers were removed via dialysis, and the final product was freeze-dried and structurally characterized by ^1^H-NMR (Figure S3).

Using NH_2_-PEG_108_-CTA as a macro-RAFT agent, NH_2_-PEG_108_-CTA (0.040 mmol) and DPA (1.114 mmol) were dissolved in anhydrous DMF, followed by the addition of AIBN (0.0019 mmol).

After three vacuum-nitrogen cycles, the reaction was carried out at 70 °C for 24 h. The resulting NH_2_-PEG_108_-PDPA_38_ product was purified via dialysis and freeze-dried. Then, NH_2_-PEG_108_-PDPA_38_ (0.001 mmol) was dissolved in anhydrous DMF, and α-D-Mannopyranosylphenyl isothiocyanate (0.005 mmol) with diisopropylethylamine (DIEA, 0.005 mmol) was added. The reaction proceeded at room temperature for 24 h. After completion, the product was dialyzed, freeze-dried, and characterized by ^1^H-NMR to confirm its chemical structure (Figure S4).

To assess polymerization control and uniformity, gel permeation chromatography (GPC) was performed (Figure S5). GPC analysis was conducted using tetrahydrofuran (THF) as the mobile phase, calibrated with polystyrene standards. The experiment utilized an Agilent 1260 Infinity GPC system (Agilent Technologies, USA), equipped with UV and refractive index (RI) detectors. Samples were dissolved in THF (1 mg/mL), filtered through a 0.22 μm PTFE membrane, and injected into the GPC column. The flow rate was set to 1.0 mL/min, and the column temperature was maintained at 35 °C. Agilent GPC/SEC software was used for data processing 39.

### Preparation and characterization of nano-vaccine

The polymeric micelle nanoparticles were prepared using the nano co-precipitation method. The diblock copolymer and PEI were dissolved in THF at the molar ratio specified in Table S1, followed by the addition of deionized water under ultrasonic agitation. The resulting micelle nanoparticles were purified by dialysis overnight against deionized water to remove THF.

For the loading of the novel antigen peptide into the nano-micelles, M32 peptide was first dissolved in DMSO at a concentration of 10 mg/mL (1 mL) and then dispersed in 2 mL of endotoxin-free water. The copolymer was dissolved in THF as described previously, and then added to the peptide solution under ultrasonic agitation. THF and unbound antigen peptides were removed via ultrafiltration for 15 min using a molecular weight cutoff of 50 kDa.

The hydrated particle size and zeta potential of the nanoparticles were measured using a Zetasizer Nano ZS 90 (Malvern Instruments, UK). The morphological characterization of nanoparticles was performed using transmission electron microscopy (TEM) (Hitachi H-7650, Shanghai Baihe Instrument Technology Co., Ltd.). For TEM imaging, the sample was placed on a carbon-coated copper grid, negatively stained with phosphotungstic acid (PTA, 2% w/v, Sigma-Aldrich, 496626) for 10 s, and then air-dried. The nanoparticle morphology was observed and captured at an accelerating voltage of 100 kV.

### Detection of ADU-S100 and M32 release from the nano-vaccine

The release behavior of ADU-S100 and M32 from dPEDE-A@M32 was evaluated using a centrifugation-based assay. Briefly, dPEDE-A@M32 was suspended in dissolution media (10 mM phosphate buffer, pH 6.0 or pH 7.4) and incubated in a thermostatic shaker at 100 rpm and 37 °C. At predetermined time intervals, samples were collected and centrifuged at 16,000 × g for 10 min. The concentration of ADU-S100 and M32 in the supernatant was analyzed using HPLC (Agilent 1260 Infinity II) 45.

### Western blot

Cells and tissues were digested with trypsin (T4799-5G, Sigma-Aldrich, USA) and lysed using a modified RIPA lysis buffer containing protease inhibitors (AR0108, Wuhan Boster Biological Technology, Wuhan, China). Protein concentrations were determined using a BCA Protein Assay Kit (AR1189, Wuhan Boster Biological Technology, Wuhan, China). Proteins were separated by SDS-PAGE and transferred onto PVDF membranes. The membranes were blocked with 5% BSA (9048-46-8, Sigma-Aldrich, USA) at room temperature for 1 hour, followed by overnight incubation at 4 °C with diluted primary antibodies (details in Table S2). After three washes with PBST (3 × 5 min each), the membranes were incubated with Anti-Mouse-HRP (Cat #7076, 1:5000; CST, USA) or Anti-Rabbit-HRP (Cat #7074, 1:5000; CST, USA) secondary antibody at room temperature for 1 hour. Following three additional PBST washes (3 × 5 min each), the membranes were treated with ECL substrate (Omt-01, Beijing Oumi Jia Medical Science and Technology, Beijing, China) and incubated for 1 minute at room temperature. Excess ECL reagent was removed, and the membranes were sealed with plastic wrap. X-ray films were exposed in a dark box for 5-10 min, then developed and fixed. The bands on the Western Blot images were quantitatively analyzed using ImageJ software, with β-actin serving as the loading control.

### *In vivo* biodistribution of nano-vaccine

To investigate the lymph node (LN) distribution of the nano-vaccine, nanoparticles formulated with Cy5.5-labeled PEDE (S34900, Thermo Fisher) and FITC-labeled M32 (F1906, Thermo Fisher) (PEDE@M32, PEDE-A@M32, dPED-A@M32, and dPEDE-A@M32) were injected at the tail base in BALB/c mice. Fluorescence images were acquired* in vivo* at designated time points using the IVIS Imaging System (PerkinElmer, USA). At the required time points, inguinal and axillary LNs along with major organs were collected for* ex vivo* fluorescence imaging. The colocalization of nanoparticles and M32 within the LNs was examined using confocal laser scanning microscopy (CLSM) on frozen LN sections.

### Detection of nanomaterial uptake

Flow cytometry (FCM) was used to assess the *in vitro* uptake of nanomaterials by cells. Freshly collected BMDCs from BALB/c mice were seeded at a density of 5 × 10^5^ cells per well in a 24-well plate and incubated overnight at 37 °C with FITC-labeled peptide nanomaterials. The intracellular fluorescence intensity of FITC was measured at designated time points (2, 4, 6, 8, and 10 h) using flow cytometer. To study the* in vivo* uptake of M32 by DCs, various nano-vaccines were administered via tail vein injection or injected subcutaneously at the base of the tail (nanocarriers: 0.05 μM/kg, M32: 2.5 mg/kg). Forty-eight h after injection, LNs were excised and digested into single-cell suspensions. The cells were then stained with anti-CD45-APC (ab210182, Abcam, UK), anti-CD11c-Alexa Fluor® 488 (ab33503, Abcam, UK), and anti-MHC-II-PE (ab93560, Abcam, UK) to detect the uptake of the M32 antigen by DCs.

### FCM analysis of DC Maturation and antigen presentation* in vitro*

To study the regulation of DC maturation and antigen presentation by nano-vaccines *in vitro*, 1 × 10^6^ BMDCs per well were stimulated with nanomaterials in a 12-well plate and incubated at 37 °C for 24 h. Except for the PBS group, cells were loaded with 20 μg/mL M32 or an equivalent concentration of 0.05 mmol/L carrier concentration. Cells were collected and DCs were isolated using anti-CD11c-FITC (BioLegend, 117306, 1:200). The cells were then stained with anti-CD80-PE (BioLegend, 104707, 1:200) and anti-CD86-APC (BioLegend, 105011, 1:200) or anti-CD40-PE/Cy5 (BioLegend, 124617, 1:200), anti-MHC-II-PE (Abcam, ab93560, UK), and anti-MHC-I-APC (BioLegend, 116517, 1:200). FCM was used to analyze the percentage of CD80⁺CD86⁺, CD40⁺, MHC-II⁺, and MHC-I⁺ positive cells in different treatment groups.

### Enzyme-linked immunosorbent assay (ELISA) for cytokine release

Serum sample collection: venous blood was allowed to clot at room temperature for 20-30 min, then centrifuged at 2000 × g for 10 min to collect the serum, which was stored at -80 °C. First, antigens were diluted to the appropriate concentration using a coating buffer. The ELISA wells were then blocked with 5% calf serum (F8318, MSK, Wuhan, China) at 37 °C for 40 min. Diluted samples were added to the ELISA wells, followed by ELISA antibodies for IL-6 (ab222503, Abcam, UK), TNF-γ (ab252363, Abcam, UK), and TNF-α (ab208348, Abcam, UK). The microtiter plates were covered with adhesive plastic and incubated at room temperature for 2 h. After incubation, the plates were read at 450 nm using a microplate reader (Bio-Rad, USA). Standard curves were generated to analyze the data.

### Nano-vaccine immunoactivation and tumor growth inhibition in animal experiments

Female BALB/c mice (randomly assigned to four groups) were administered different formulations on days 0, 7, and 14 to induce an immune response. On day 21, peripheral blood, inguinal LNs (iLNs), and spleens were collected from the treated mice to assess the immune response. The animal groups were as follows: 1) PBS group: intravenous tail injection of PBS solution on days 0, 7, and 14; 2) dPEDE group: intravenous tail injection of dPEDE blank carrier solution at 0.5 μM/kg on days 0, 7, and 14; 3) M32+A group: intravenous tail injection of a mixture of M32 and ADU-S100 solution on days 0, 7, and 14; 4) dPEDE-A@M32 group: intravenous tail injection of the dPEDE-A@M32 Nano-vaccine solution on days 0, 7, and 14. Peripheral blood was coagulated at 37 °C for 2 h and then refrigerated overnight at 4 °C. Subsequently, the blood samples were centrifuged at 3,000 g for 10 min at 4 °C to collect serum. Serum TNF-α and IFN-γ levels were measured using ELISA. To assess DC maturation in the iLNs, the adjacent LNs on the treated side were ground into a single-cell suspension, and FCM analysis was performed using the same staining method as that used for *in vitro* DC maturation and antigen presentation levels. The splenic single-cell suspension was seeded at a concentration of 2 × 10^5^ cells per well in a 96-well plate.

Female BALB/c mice (randomly assigned to four groups) were subjected to different treatments on days -21, -14, and -7. On day 0, 4T1 cells (2 × 10^6^) were subcutaneously injected into the right lower mammary fat pad to establish an immune-suppressed tumor model 46. The animal groups were as follows: 1) PBS group: Mice received tail vein injections of PBS solution on days -21, -14, and -7, followed by subcutaneous injection of 4T1 cells (2 × 10^6^) into the right lower mammary fat pad on day 0; 2) dPEDE group: Mice received tail vein injections of dPEDE blank carrier solution (0.5 μM/kg) on days -21, -14, and -7, followed by subcutaneous injection of 4T1 cells (2 × 10^6^) into the right lower mammary fat pad on day 0; 3) M32+A group: Mice received tail vein injections of M32 and ADU-S100 mixed solution on days -21, -14, and -7, followed by subcutaneous injection of 4T1 cells (2 × 10^6^) into the right lower mammary fat pad on day 0; 4) dPEDE-A group: On days -21, -14, and -7, mice received tail vein injections of dPEDE-A nano-vaccine solution. 5) dPEDE-A@M32 group: Mice received tail vein injections of dPEDE-A@M32 nano-vaccine solution on days -21, -14, and -7, followed by subcutaneous injection of 4T1 cells (2 × 10^6^) into the right lower mammary fat pad on day 0. The bioluminescent signal of 4T1 cells was analyzed using the IVIS Lumina Series III* in vivo* imaging system (PerkinElmer, USA) 47. Tumor size was measured daily with calipers, and the tumor volume was calculated using the formula: volume (mm^3^) = length × width^2^ / 2. Tumor size and survival status of each mouse were recorded every four days. On day 28 post-4T1 cell injection, the immune profile of tumor tissues and iLN was analyzed. Peripheral blood was collected from mice, and serum was isolated for TNF-α quantification analysis using ELISA. Single-cell suspensions from tumor tissues and iLN were prepared for FCM analysis 48.

### Animal experiment on nanovaccine for inhibiting tumor recurrence

To investigate the ability of the nano-vaccine to inhibit postoperative tumor regrowth, 1 × 10^6^ 4T1-luc cancer cells were implanted into the right mammary fat pad of 6- to 8-week-old female BALB/c mice. On day 10, the tumors were surgically removed, leaving approximately 1% of the tumor tissue to simulate postoperative residual micro-tumors. Animal Grouping: 1) PBS Group: Tail vein injections of PBS solution were administered on days 10, 13, 16, and 19; 2) dPEDE Group: Tail vein injections of dPEDE blank carrier solution (0.5 μM/kg) were administered on days 10, 13, 16, and 19; 3) M32+A Group: Tail vein injections of a mixture of M32 and ADU-S100 were administered on days 10, 13, 16, and 19; 4) dPEDE-A group: On days 10, 13, 16, and 19, mice received tail vein injections of dPEDE-A nano-vaccine solution; 5) dPEDE-A@M32 Group: Tail vein injections of dPEDE-A@M32 nano-vaccine solution were administered on days 10, 13, 16, and 19. Tumor volume in recurrent tumors was measured every four days using calipers, and survival status was recorded. On days 10, 11, 17, and 22, bioluminescence imaging of tumors was performed using an *in vivo* imaging system, and data were analyzed with *in vivo* imaging software (PerkinElmer, USA) 49.

### Animal experiment demonstrating inhibition of systemic bloodstream metastasis by nano-vaccine

1 × 10^6^ 4T1-luc cancer cells were implanted into the left mammary fat pad of BALB/c mice. To simulate more aggressive invasion and hematogenous metastasis, 5 × 10^5^ 4T1-luc cells were injected intravenously into the mice on day 10. Animal grouping was as follows: 1) PBS Group: Tail vein injections of PBS solution were administered on days 7, 10, 13, and 16; 2) dPEDE Group: Tail vein injections of dPEDE blank carrier solution (0.5 μM/kg) were administered on days 7, 10, 13, and 16; 3) M32+A Group: Tail vein injections of a mixed solution of M32 and ADU-S100 were administered on days 7, 10, 13, and 16; 4) dPEDE-A group: On days 7, 10, 13, and 16, mice received tail vein injections of dPEDE-A nano-vaccine solution; 5) dPEDE-A@M32 Group: Tail vein injections of dPEDE-A@M32 nano-vaccine solution were administered on days 7, 10, 13, and 16. On day 20, six mice from each group were euthanized, and fresh lungs were collected. Tumor metastasis in the lungs was monitored using *in vitro* bioluminescence imaging. Lung and liver sections were subjected to Hematoxylin and Eosin (H&E) staining to evaluate anti-metastatic effects. The metastatic area in the sections was quantified using ImageJ software. For the remaining six mice in each group, tumor size and survival rate were recorded 49.

### Animal experiment of nano-vaccine and immune checkpoint blockade (ICB) combination therapy

1 × 10^6^ 4T1-luc cancer cells were implanted into the left mammary fat pad of BALB/c mice. On days 7, 14, and 21, the mice received dPEDE-A@M32 vaccine injections, and on days 8, 15, and 22, they received αPD-1 treatment. Tumor size was measured daily using calipers, and the tumor volume was calculated using the formula: volume (mm^3^) = length × width^2^ / 2. When the tumor size reached 200 mm^3^, the mice were randomly divided into the following treatment groups: 1) PBS Group (Control group, received equivalent volume of PBS via tail vein injection); 2) anti-PD-1 Group: Intraperitoneal injections of 200 μg anti-PD-1 (Clone: RMP1-14, BioXcell) on days 8, 15, and 22; 3) dPEDE-A@M32 Group: Tail vein injections of dPEDE-A@M32 (0.5 μM/kg) on days 7, 14, and 21; 4) dPEDE-A@M32 + anti-PD-1 Group: Tail vein injections of dPEDE-A@M32 on days 7, 14, and 21, combined with intraperitoneal injections of anti-PD-1 on days 8, 15, and 22. On day 28, six mice from each group were euthanized by intraperitoneal injection of an overdose of pentobarbital sodium (100 mg/kg). The tumors were then excised and weighed 50.

### Immunofluorescence

For cellular immunofluorescence staining, cells were counted and plated in immunofluorescence chambers at a density of 2 × 10^5^ cells per well. Once the cells reached approximately 90% confluence, they were fixed with 4% paraformaldehyde (P885233, Macklin, USA) for 15-30 min. For tissue immunofluorescence, tissues were fixed in 4% paraformaldehyde, processed into 4 μm paraffin sections, deparaffinized, and rehydrated, following standard immunohistochemical staining protocols. Subsequently, the samples were treated with 0.1% Triton (L885651, Macklin, USA) for 15 min. After two washes with PBS, the samples were incubated overnight at 5 °C with PBS containing 15% FBS. The cells or tissues were then incubated overnight at 4 °C with primary antibodies F4/80 (Abcam, ab100790, 1:200), CD8 (MA5-29682, 1:100, Thermo Fisher, USA), IFN-γ (14-7313-81, 1:200, Thermo Fisher, USA), and PD-1 (Abcam, ab214421, 1:200). After three washes with TBST (1% Tween-20 in TBS), the samples were incubated at room temperature for 2 h with secondary antibodies: goat anti-rabbit Alexa Fluor® 568 conjugate (A-11011, Thermo Fisher, USA) or goat anti-rabbit Alexa Fluor™ 594 conjugate (A-11012, Thermo Fisher, USA). Finally, the samples were counterstained with DAPI (D1306, Thermo Fisher, USA) and observed under a fluorescence microscope (Zeiss Observer Z1, Germany). Fluorescence intensity in selected target areas was measured and analyzed quantitatively using ImageJ, and the percentage of positive cells was statistically calculated.

### Immunohistochemistry

The tissues to be tested were fixed and embedded. The embedded tissues were then sectioned into thin slices, deparaffinized to remove wax, and rendered hydrophilic for subsequent immunostaining. The deparaffinized sections were treated with a specific Ki67 antibody (SAB5700770, 1:200; Sigma Aldrich, USA) and an Anti-Rabbit-HRP secondary antibody (12-348, 1:1000; Sigma Aldrich, USA). The sections were then stained using the DAB substrate kit (ab64238, Abcam, USA) to visualize the antibody binding sites. After staining, the sections were re-dehydrated and mounted for observation. The stained sections were examined under a microscope, and expression levels were recorded. The staining results were evaluated by randomly selecting five lesion areas under the microscope and calculating the percentage of positively stained cells.

### Multicolor FCM

Multicolor FCM was used to detect the composition of immune cells in the subcutaneous xenograft tumor model. Tumor samples from each group of mice were collected and digested in HBSS solution (ThermoFisher Scientific, 24020117) containing 0.5 mg/mL type IV collagenase and 0.25 mg/mL DNase I at 37°C for 30 min. The digested samples were then filtered through a 40 μm cell strainer and centrifuged at 400 g for 10 min to obtain a single-cell suspension.

To minimize nonspecific binding, the cells were incubated with Fc receptor-blocking antibody (BioLegend, 101320, USA) for 15 min. The cells were then stained with fluorescently labeled monoclonal antibody mixtures, including CD11b-PE (BioLegend, 101208), CD11c-FITC (BioLegend, 117306), and CD3-APC (BioLegend, 100236) for detecting basic immune cell subsets; F4/80-APC (BioLegend, 123116) and CD206-FITC (BioLegend, 141704) for identifying CD11b⁺ macrophage subsets; CD80-PE (BioLegend, 104707) and CD86-APC (BioLegend, 105011) for detecting CD11c⁺ dendritic cell subsets; CD4-PerCP (BioLegend, 100432) and CD8-PE (BioLegend, 100708) for distinguishing CD3⁺ T cell subsets; Foxp3-APC (BioLegend, 126404), CD62L-FITC (BioLegend, 161212), and CD44-BV510 (BioLegend, 103044) for classifying CD3⁺CD4⁺ and CD3⁺CD8⁺ T cell subsets. All antibodies were purchased from BioLegend, USA, and diluted at 1:200. The samples were incubated at 4°C in the dark for 30 min, washed twice with PBS (containing 2% FBS) to remove unbound antibodies, and finally resuspended in 500 μL PBS.

Data acquisition was performed using a BD FACSCanto II flow cytometer, and FlowJo v.10 software (FlowJo LLC) was used for analysis. The analysis strategy involved first setting gates on FSC/SSC plots to select the lymphocyte population. Next, a single-cell gate (FSC-H vs. FSC-A) was established to exclude debris and cell aggregates. T cells and myeloid cells were then distinguished based on CD3 and CD11b/CD11c expression. Further gating was applied to analyze CD4⁺ and CD8⁺ T cells, classifying them into naïve (CD62L⁺CD44⁻), effector memory (CD62L⁻CD44⁺), and regulatory (Foxp3⁺CD4⁺) T cells. Macrophage subsets were identified based on CD11b and F4/80 expression and further distinguished into M1 (CD80⁺) and M2 (CD206⁺) macrophages. Dendritic cell subsets were defined using CD11c gating, and their maturation status was assessed based on CD80⁺CD86⁺ expression.

### Biochemical parameter detection

The metabolic parameters in the serum of mice from each group were measured using the ALT Activity Assay Kit (E1010, Sigma-Aldrich, USA), AST Activity Assay Kit (E1020, Sigma-Aldrich, USA), BUN Assay Kit (60-1100, BioAssay Systems, USA), and Creatinine Assay Kit (CR200, Randox Laboratories Ltd, UK). The procedures were carried out according to the manufacturer's instructions provided with each kit.

### Histopathological staining

Cell apoptosis in paraffin-embedded tissue sections was assessed using the TUNEL Staining Kit. Tumor tissues were sectioned into 6 μm slices, deparaffinized, and rehydrated. The sections were then incubated in Tris buffer containing 15.3 mg/mL proteinase K (pH 8) at room temperature for 20 min, followed by a wash with 50 mM TBS (pH 7.6). Next, the sections were treated with a green fluorescent enzyme solution (C1086, Beyotime, Shanghai, China). After TUNEL labeling, nuclei were stained with DAPI. Apoptotic cells, exhibiting green fluorescence, were observed using CLSM, and the percentage of TUNEL-positive cells was quantified using ImageJ.

H&E staining: Tissue samples were collected, fixed, and sectioned. The paraffin sections were deparaffinized in xylene and rehydrated through a graded ethanol series (100%, 95%, and 70%) followed by washing in water. The sections were stained with hematoxylin solution (H8070, Solarbio, Beijing, China) at room temperature for 5-10 min. After washing with distilled water, the sections were dehydrated in 95% ethanol and stained with eosin solution (G1100, Solarbio, Beijing, China) for 5-10 min. The sections were then dehydrated, cleared, and mounted using standard protocols.

### Statistical analysis

Data were collected from at least three independent experiments and are presented as mean ± standard deviation (SD). For comparisons between two groups, an independent samples t-tests were performed. For comparisons involving three or more groups, a one-way analysis of variance (ANOVA) was conducted. If ANOVA indicated significant differences, Tukey's Honestly Significant Difference (HSD) post hoc test was applied to compare differences between groups. For non-normally distributed or inhomogeneous variance data, Mann-Whitney U test or Kruskal-Wallis H test was used. All statistical analyses were performed using GraphPad Prism 9 (GraphPad Software, Inc.). A significance level of 0.05 was set for all tests, and *p*-values < 0.05 were considered statistically significant.

## Results

### Successful development of a pH-responsive nano-vaccine for targeted delivery of STING agonist and neoantigen in BC treatment

BC is among the most prevalent malignancies in women worldwide. Traditional treatments include surgery, radiotherapy, chemotherapy, and hormone therapy 51, 52. With advances in understanding cancer biology, new therapeutic approaches, such as targeted and immunotherapies, have gained increasing significance 53, 54. Neoantigen-based vaccines activate tumor-specific cytotoxic T lymphocytes (CTLs), generating robust immune responses against specific tumor antigens and continuously targeting cancer cells 55, 56. Despite challenges in antigen processing and presentation, such as DC activation issues, studies indicate that STING pathway activation is crucial for enhancing immune responses and maintaining immune surveillance 57, 58. Therefore, developing immunotherapeutic vaccines for BC not only enables more personalized treatment but also mitigates the side effects of conventional therapies, improving patients' quality of life and survival rates, highlighting the necessity and potential of BC vaccines 59.

**Figure 1:**
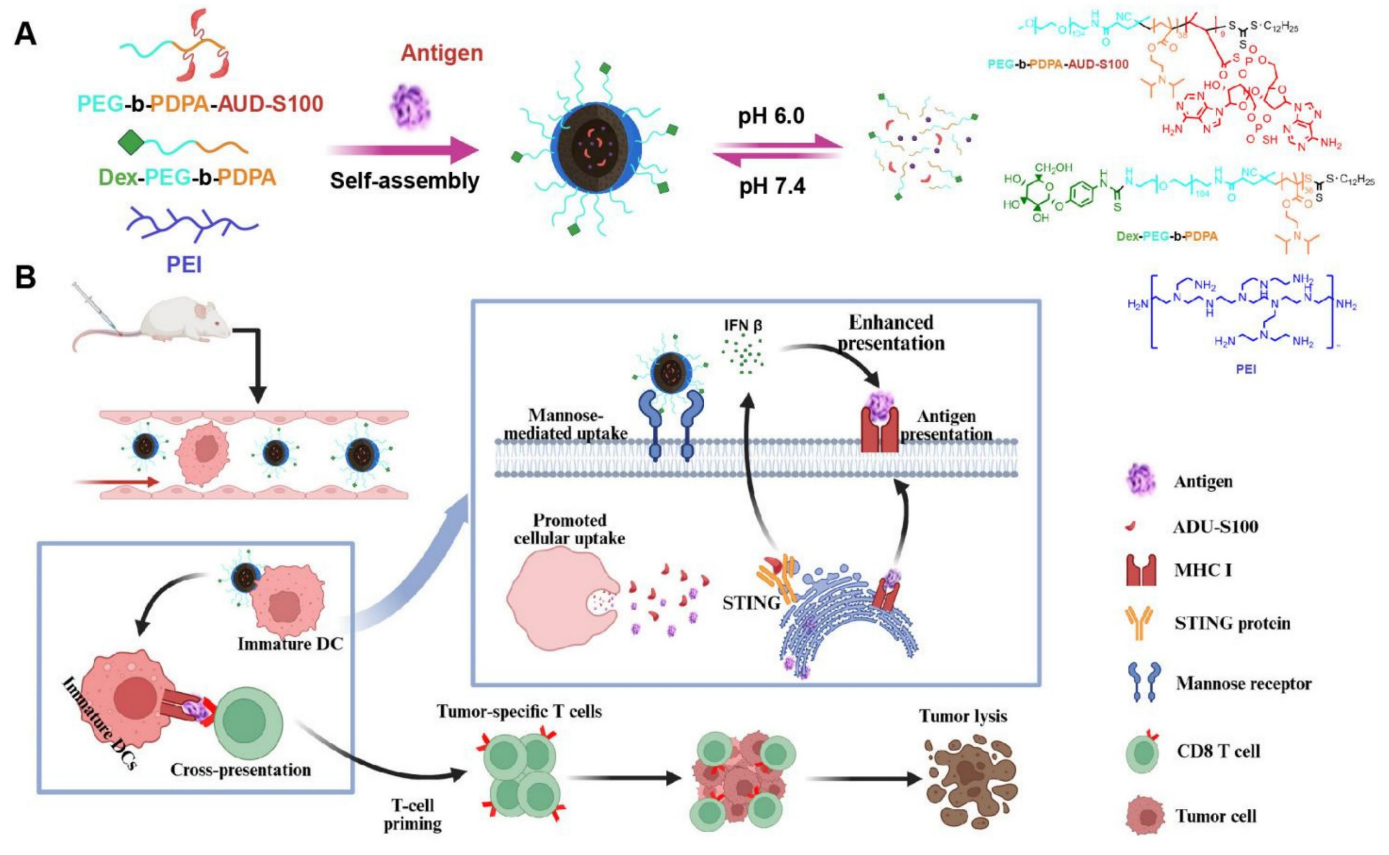
** Schematic of the nano-vaccine delivering STING agonist and antigen.** Note: (A) Schematic illustration of the nano-vaccine preparation and disassembly process of the nano-vaccine; (B) Diagram illustrating how the nano-vaccine enhances the STING pathway and boosts T-cell immune responses to improve immunity.

This study presents a nanovaccine targeting the STING pathway for personalized immunotherapy in BC. The nanovaccine consists of a pH-responsive amphiphilic polymer, the neoantigen M32, and the STING agonist ADU-S100 (Figure 1A). These nanoparticles exploit their inherent intracellular escape mechanisms to facilitate the release of neoantigens into the cytoplasm. The STING agonist triggers STING pathway activation in DCs, inducing the secretion of type I interferons (IFNs), which promote T-cell activation. Concurrently, the nanovaccine integrates neoantigens to stimulate a tumor-specific T-cell immune response (Figure 1B).

**Figure 2:**
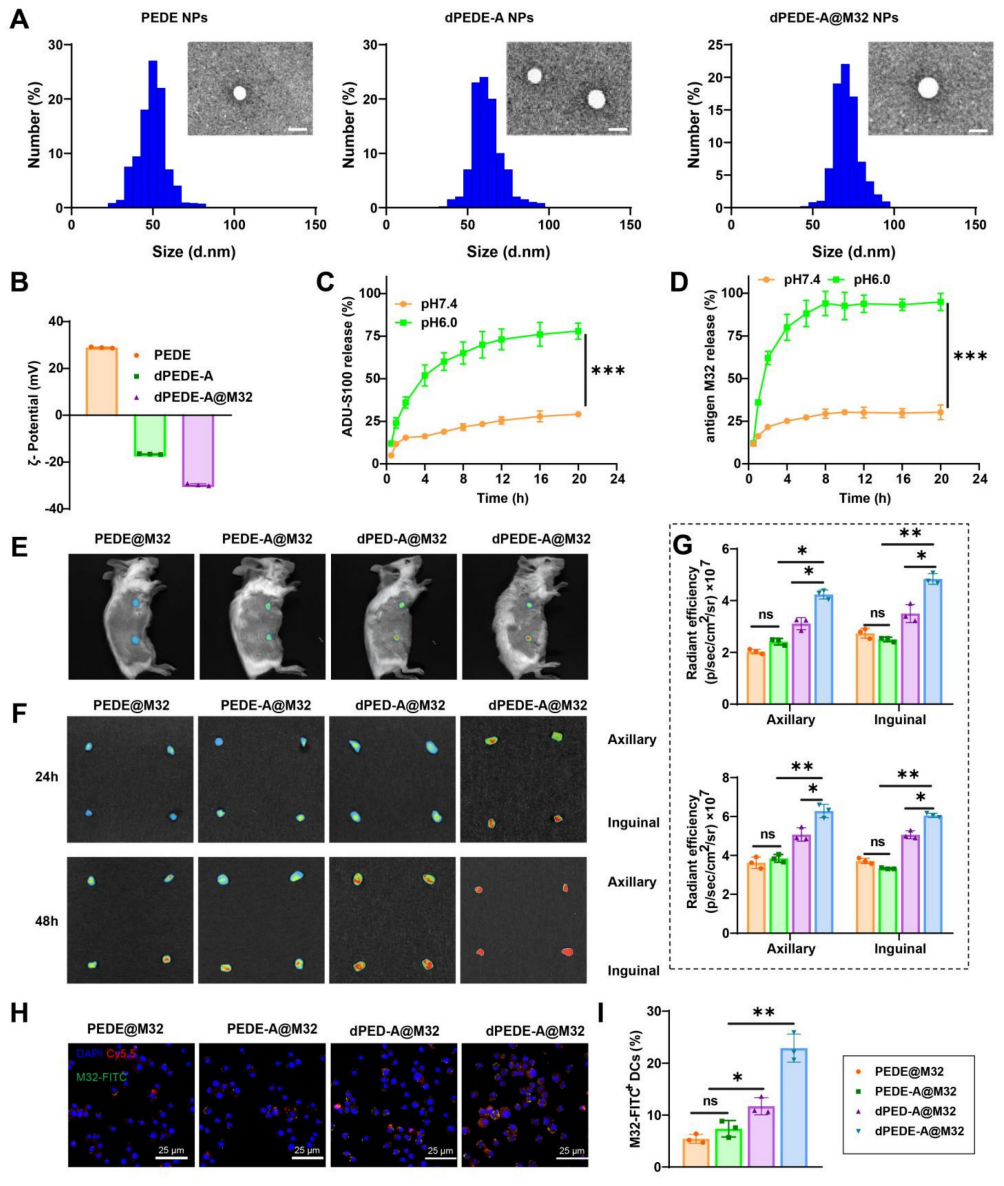
** Schematic of the nano-vaccine delivering STING agonist and antigen.** Note: (A) DLS and TEM images (top left) showing the size distribution and morphology of PEDE, dPEDE-A, and dPEDE-A@M32 nano-vaccines, scale bar = 50nm; (B) Zeta potential measurement of PEDE, dPEDE-A, and dPEDE-A@M32 nano-vaccine; (C) Release curve of ADU-S100 from dPEDE-A@M32 at different pH conditions; (D) Release curve of the model antigen M32 from dPEDE-A@M32 at different pH conditions; (E) Representative* in vivo* fluorescence imaging showing the biodistribution of PEDE@M32, PEDE-A@M32, dPED-A@M32, and dPEDE-A@M32 24 h after subcutaneous injection in mice; (F-G) Fluorescence imaging of axillary and iLNs isolated 24 or 48 h after subcutaneous injection (F) and corresponding statistical analysis (G) of axillary and inguinal LNs at 24 or 48 h after subcutaneous injection, assessing the biodistribution of PEDE@M32, PEDE-A@M32, dPED-A@M32, and dPEDE-A@M32 *in vivo*; (H) CLSM detection of co-localization of antigen and micelle nanoparticles in iLNs 48 h after injection, bar = 25μm; (I) Fluorescence labeling detection of DC (CD45^+^CD11c^+^MHCII^+^) uptake of M32 48 h post-inoculation. *In vitro* experiments were repeated three times, with three animals per group in animal experiments. Values are presented as mean ± SD. 'ns' indicates no significant difference between groups, * *p* < 0.05, ** *p* < 0.01, *** p < 0.001.

The nano-vaccine was self-assembled in the presence of the M32 antigen peptide using one or more of the following amphiphilic molecular chains: PEG-b-PDPA, dex-PEG-b-PDPA, PEG-b-PDPA-ADU-S100, and the cationic polymer PEI. Studies have shown that PEI's positive charge enhances electrostatic interactions with negatively charged cell membranes, promoting endocytosis and significantly improving DC uptake of nanoparticles 60-63. Additionally, PEI enhances DC stimulation in murine models, inducing tumor-targeted cytotoxic T-cell responses 62, 63. TEM analysis confirmed that the PEDE, dPEDE-A, and dPEDE-A@M32 exhibited spherical morphology (Figure 2A). Dynamic light scattering (DLS) revealed particle diameters of 51.6 ± 4.1 nm for PEDE, 59.2 ± 6.7 nm for dPEDE-A, and 71.5 ± 5.9 nm for dPEDE-A@M32 (Figure 2A). Following antigen loading, the nanoparticles' zeta potential shifted from positive to negative (Figure 2B). The release of ADU-S100 from dPEDE-A@M32 in acidic media reached 78% within 20 h, compared to only 27% under neutral conditions (Figure 2C). The antigen peptide showed a similar release profile, with 76.2% released within 10 h in acidic conditions and only 24.5% under neutral conditions (Figure 2D).

To investigate the *in vivo* biodistribution of the nano-vaccine, the blank carrier was labeled with Cy5.5, while the antigen was labeled with FITC, and the formulation was administered to BALB/c mice via tail vein injection. *In vivo* fluorescence imaging showed that 24 h post-injection, the dPEDE-A@M32 nano-vaccine efficiently accumulated in the LNs (Figure 2E). *In vitro* fluorescence imaging of major organs revealed significant enrichment of the nano-vaccine in the axillary LNs, with minimal presence in major organs such as the heart, liver, spleen, lungs, and kidneys (Figure 2F, Figure S6A-B). The targeting efficiency of dPEDE-A@M32 to LNs peaked at 24 and 48 h post-injection. CLSM examination 48 h post-injection revealed minimal free M32 distribution in the LNs (Figure S6B). In contrast, the nano-vaccine efficiently delivered antigens to the LNs. Notably, the dPEDE-A@M32 group showed higher accumulation in the LNs than the dPED-A@M32 group, benefiting from the transmembrane action of the cationic polymer PEI, resulting in a more diffuse accumulation pattern (Figure 2F-G). At 48 h post-administration, fluorescence intensity measurements of antigen-presenting cells (APCs) uptake of nanoparticles and antigen indicated significantly higher fluorescence intensity in the dPEDE-A@M32 group compared to other groups (Figure 2H-I). Previous studies suggest that LN-targeted nanoparticles enhance T-cell activation and exert anti-tumor effects while also preventing tumor invasion of lymphatic vessels 64. Additionally, nanomaterials facilitate the targeted delivery of immunomodulators to tumors and lymphoid organs, modulating the interaction of biological agents with immune cells and promoting their accumulation in tumors and bone marrow-derived immune cells within systemic compartments 65. These findings support the hypothesis that nanomedicines can enhance antigen presentation in LNs, optimizing T-cell activation and boosting anti-tumor immune responses. H&E analysis of the main organs (heart, liver, spleen, lungs, kidneys) of the mice post-nanoparticle injection showed no histological damage (Figure S7A). Serum markers for liver (aspartate aminotransferase (AST), alanine aminotransferase (ALT)) and kidney function (blood urea nitrogen (BUN), creatinine (CR)) remained within normal levels (Figure S7B), indicating that the nano-vaccine exhibits no significant toxicity and preserves normal hepatic and renal function.

The results confirm the successful development of the pH-responsive nano-vaccine, dPEDE-A@M32, which enables the simultaneous delivery of a STING agonist and a neoantigen. This nano-vaccine exhibits excellent lymphatic targeting capabilities and holds promise for cancer immunoprevention.

### Induction of DC maturation and enhanced antigen presentation by nano-vaccine activating the cGAS-STING pathway and upregulating immune regulatory signals

The efficiency of antigen uptake by DCs is a key parameter for evaluating antigen delivery effectiveness 66-68. Therefore, cellular uptake analyses were conducted to assess the nano-vaccine's delivery efficiency in DCs. BMDCs were co-incubated with M32-FITC+ADU-S100, blank carrier dPEDE-FITC, and nano-vaccine dPEDE-A@M32-FITC, referred to as M32+A, dPEDE, and dPEDE-A@M32 respectively. FCM analysis was performed after 1, 4, and 12 h of co-culture. Results indicated that, compared to PBS, the FITC-labeled M32 fluorescence intensity was significantly higher in the dPEDE-A@M32 group, with the highest percentage of FITC-positive cells. Interestingly, in the dPEDE-A@M32 group, the FITC signal at 12 h was lower than at 4 h, likely due to the metabolic degradation of M32-FITC in DCs. The fluorescence signal of free M32-FITC+A increased over time, but it remained significantly lower than that of dPEDE-A@M32 (Figure S8A-B).

**Figure 3:**
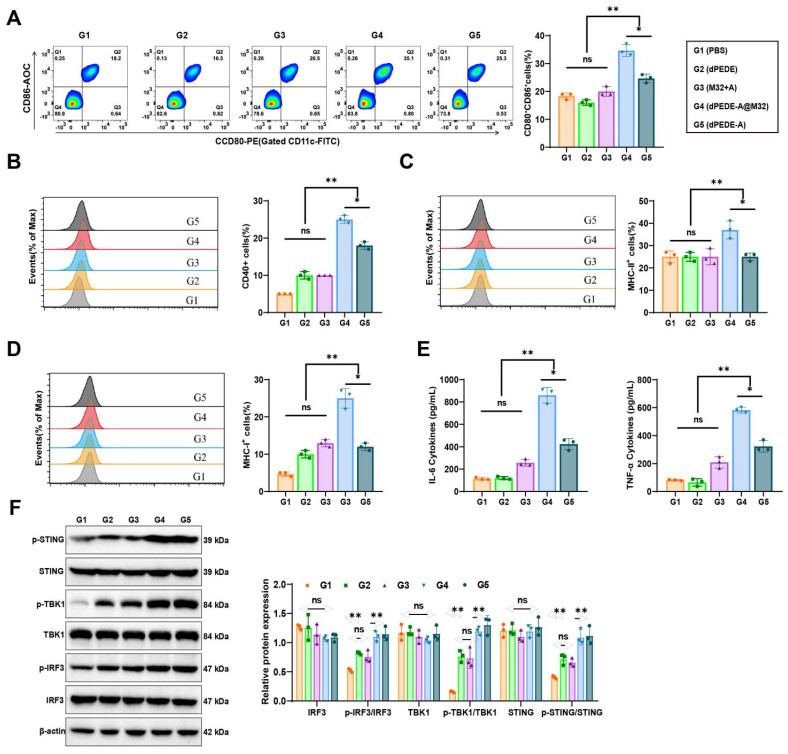
** Experimental validation of dPEDE-A@M32 in promoting DC maturation and antigen cross-presentation while activating the cGAS-STING pathway.** Note: (A) FCM analysis of BMDCs co-incubated for 24 h with free M32-FITC+ADU-S100, blank carrier dPEDE-FITC, and nano-vaccine dPEDE-A@M32-FITC, showing the percentage of CD80⁺CD86⁺ positive cells; (B-D) FCM analysis of BMDCs after 24-hour co-incubation with free M32-FITC+ADU-S100, blank carrier dPEDE-FITC, and nano-vaccine dPEDE-A@M32-FITC, (B) Percentage of CD40-positive cells, (C) Percentage of MHC-II-positive cells, (D) Percentage of MHC-I-positive cells; (E) ELISA measurements of IL-6 and TNFα levels in the supernatants of BMDCs from different treatment groups; (F) Western Blot analysis detecting the protein expression levels of p-STING, p-TBK1, and p-IRF3 in BMDCs under different treatment groups. *In vitro* experiments were repeated three times. Values are presented as mean ± SD. 'ns' indicates no significant difference between groups, * *p* < 0.05, ** *p* < 0.01, *** p < 0.001.

Mature DCs exhibit upregulation of co-stimulatory molecules and release pro-inflammatory cytokines 69, 68. The interaction between DCs and T-cells is crucial as it governs the interplay and communication between these two cell types 70. FCM analysis of BMDCs treated under various conditions demonstrated that co-culturing with dPEDE-A or dPEDE-A@M32 resulted in a significant upregulation of co-stimulatory molecules (CD80, CD86, and CD40), with the highest expression observed in the dPEDE-A@M32 group (Figure 3A-B). CD80, CD86, and CD40 are essential co-stimulatory molecules in BMDC-T cell interactions. The FCM analysis indicated that treatment with dPEDE-A@M32 resulted in higher MHC-II expression levels on DCs, consistent with the enhanced uptake of M32 observed in this group (Figure 3C). This upregulation suggests a potent adjuvant effect of dPEDE.

DCs capture the M32 antigen from the nano-vaccine, which is subsequently cleaved into peptides. These peptides bind to MHC-I molecules on the surface of DCs, leading to the presentation of the MHC-I (H-2Kb)-bound SIINFEKL complex. Interaction between CD8 T cells and the antigen-MHC-I complex triggers activation, proliferation, and differentiation into CTLs. FCM analysis revealed a significant upregulation of MHC-I positive cells in the BMDCs of the dPEDE-A@M32 group compared to the M32+A, dPEDE, dPEDE-A groups (Figure 3D). Furthermore, BMDCs treated with dPEDE-A@M32 secreted higher levels of IL-6 and TNF-α (Figure 3E). The secretion of these cytokines promotes DC activation and maturation, enhancing their migration to LNs for T-cell interactions 71, 72.

ADU-S100 is a STING agonist 73, and dPEDE-A@M32 is hypothesized to activate the STING pathway. Western Blot analysis of key proteins in the signaling pathways across different treatment groups revealed phosphorylation and activation of TBK1 and IRF3, both components of the STING pathway, which drive the transcriptional activation of IFNs and IFN-stimulated genes (ISGs) 74. In the dPEDE-A and dPEDE-A@M32 treatment group, phosphorylated STING, TBK1, and IRF3 levels were significantly elevated, whereas total STING, TBK1, and IRF3 levels remained unchanged, confirming activation of the cGAS-STING pathway (Figure 3F).

These results demonstrate that dPEDE-A@M32 induces DC maturation, enhances antigen presentation, and activates the cGAS-STING pathway, leading to the upregulation of downstream immune regulatory signals.

### Enhancement of Murine DC Immune Responses and Tumor Growth Inhibition by Nano-vaccine

**Figure 4:**
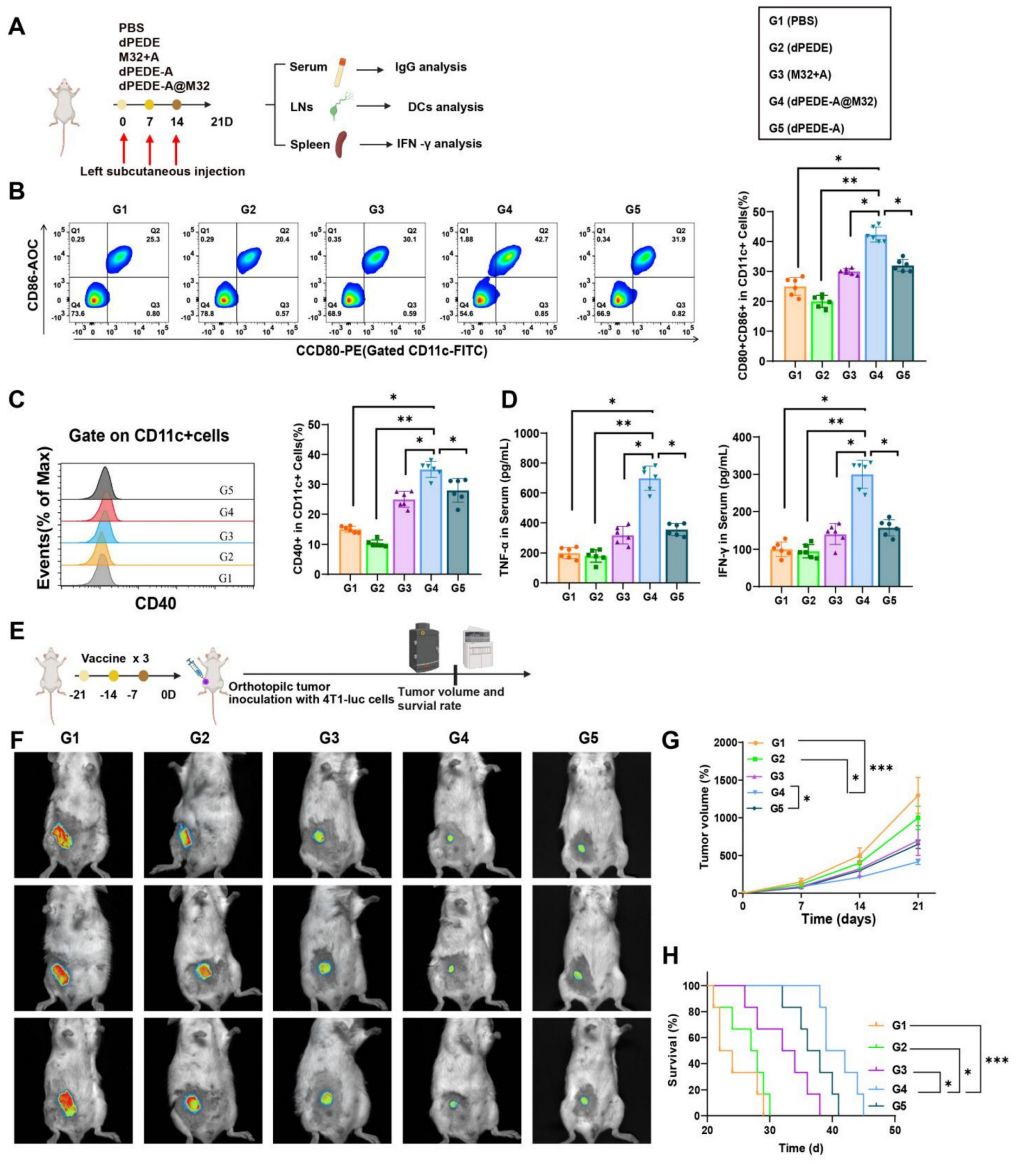
** Impact of nano-vaccine mediated mouse DC immune responses on primary tumor development.** Note: Mice were divided into four groups and received subcutaneous injections of PBS, dPEDE, M32+A, dPEDE-A, and dPEDE-A@M32 on days 0, 7, and 14 in the left flank. On day 21, serum, splenocytes, and iLNs cells were collected to assess the immunological effects of the nano-vaccine. (A) Schematic diagram of the mouse immune model and experimental procedure; (B) FCM analysis of the proportion of CD80^+^CD86^+^ positive DCs extracted from the iLNs of mice in different treatment groups; (C) Percentage of CD40^+^ positive DCs extracted from the iLNs of mice in different treatment groups; (D) ELISA measurements of TNF-α and IFN-γ levels in the serum of mice from different treatment groups; (E) Schematic diagram of the mouse immune tumor growth model and experimental procedure; (F) *In vivo* bioluminescence imaging of 4T1-luc tumors, showing three representative mice per group; (G) Tumor growth curves for different treatment groups; (H) Survival curves for different treatment groups. Each group in animal experiments consisted of six mice, and values are presented as mean ± SD, ** *p* < 0.01, *** *p* < 0.001.

To investigate the nano-vaccine's ability to induce antigen-specific humoral and cellular immune responses *in vivo*, mice were divided into four groups and subcutaneously injected on the left side with PBS, dPEDE, M32+A, dPEDE-A, and dPEDE-A@M32 on days 0, 7, and 14. On day 21, serum, splenocytes, and iLNs were collected to assess the vaccine's immunological effects (Figure 4A). In DCs collected from iLN cells, the proportion of co-stimulatory molecules CD80^+^CD86^+^ and CD40^+^ in the dPEDE-A@M32 treatment group was significantly higher than in the control groups (Figure 4B-C). This indicates that dPEDE-A@M32 more effectively activates DCs, promoting their maturation and antigen presentation, consistent with the *in vitro* findings.

Additionally, serum levels of TNF-α and IFN-γ in mice treated with dPEDE-A@M32 were significantly elevated, indicating a robust cell-mediated immune response against intracellular pathogens (Figure 4D). To further evaluate its therapeutic potential, a 4T1 transplant model was established using mice immunized with three doses (Figure 4E). Tumor growth curves and mouse survival curves demonstrated reduced tumor progression and prolonged survival in the dPEDE-A@M32 group (Figure 4F-H).

These results illustrate that nano-vaccine immunization enhances DC responses in mice, effectively inhibiting tumor growth.

### Nano-vaccine dPEDE-A@M32 Promotes Immune Response and Inhibits Tumor Recurrence in a BC Model

**Figure 5:**
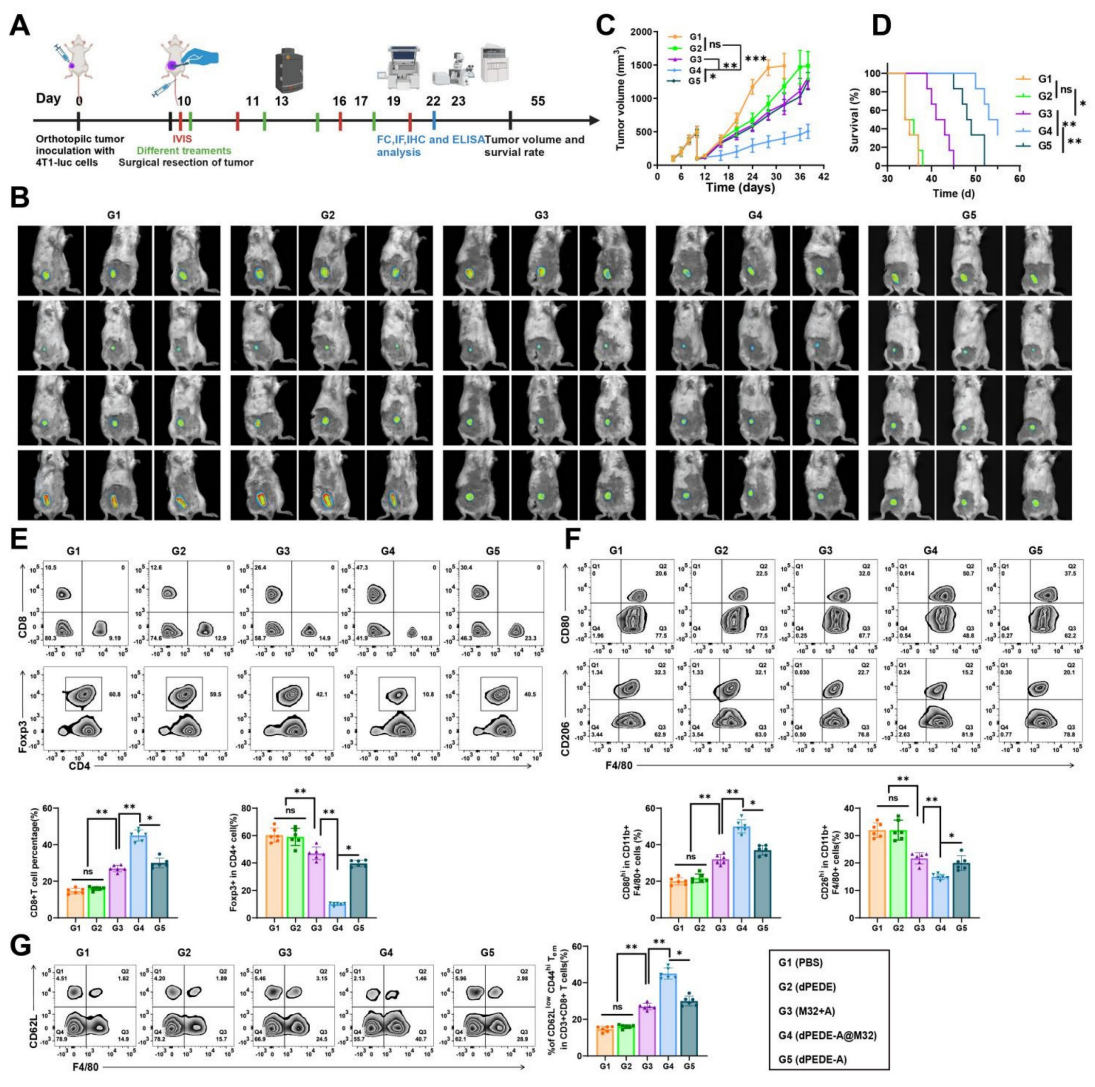
** Observing the therapeutic efficacy of nano-vaccine on BC recurrence.** Note: (A) Schematic diagram of treatment in the 4T1-luc orthotopic tumor incomplete resection model; (B) *In vivo* bioluminescence imaging of 4T1-luc tumors post-primary tumor resection, displaying three representative mice per group; (C) Tumor growth curves of the tumor resection model treated with PBS, dPEDE, M32+A, dPEDE-A, and dPEDE-A@M32; (D) Survival curves of the tumor resection model treated with PBS, dPEDE, M32+A, dPEDE-A, and dPEDE-A@M32; (E) FCM analysis of tumor-infiltrating CD8^+^ T cells and CD4^+^Foxp3^+^ Tregs, with images and relative quantitative statistics; (F) FCM analysis of TAMs: M1 type (CD80^hi^ CD11b^+^ F4/80^+^) and M2 type (CD206^hi^ CD11b^+^ F4/80^+^), with images and relative quantitative statistics; (G) FCM analysis of effector memory T-cells (CD62L^low^ CD44^hi^ CD3^+^ CD8^+^ TEM) in the spleen, with images and relative quantitative statistics. Each group in animal experiments consisted of six mice, and values are presented as mean ± SD. 'ns' indicates no significant difference between groups, * *p* < 0.05, ** *p* <0.01, *** *p* < 0.001.

Despite advancements in surgical techniques, tumor recurrence remains a clinical challenge due to residual and circulating tumor cells 75. Considering the nano-vaccine's capacity to stimulate anti-tumor immunity, we established a BC resection model to investigate its anti-recurrence effects (Figure 5A). Tumors were resected, leaving approximately 1% of the mass to mimic post-surgical residual microtumors. Tumor recurrence was monitored via bioluminescence signals from 4T1-luc cells and recurring tumor volumes were measured with calipers. While the M32+A or dPEDE-A treatment alone failed to suppress tumor recurrence and showed poor survival rates, the dPEDE-A@M32 treatment significantly reduced bioluminescence intensity, delayed tumor recurrence, and improved long-term survival (Figure 5B-D). Subsequently, we harvested the residual tumors treated differently and analyzed tumor-infiltrating lymphocytes (TILs) using FCM. Compared to other controls, dPEDE-A@M32 treatment notably increased CD8^+^ T-cell infiltration while reducing CD4^+^ Foxp3^+^ Tregs (Figure 5E). Additionally, FCM results showed a decrease in the percentage of M2-like TAMs and an increase in M1-like TAMs following treatment with dPEDE-A@M32 (Figure 5F). The phenotypic polarization from M2-like to M1-like TAMs can block TAM-mediated tumor angiogenesis and lymphangiogenesis, ultimately inhibiting tumor metastasis and recurrence 76, 77. Furthermore, we monitored differences in the percentage of effector memory T-cells in secondary lymphoid organs (spleen), where dPEDE-A@M32 treatment significantly elevated effector memory T-cells levels, confirming the nano-vaccine's ability to induce immune memory (Figure 5G).

These findings suggest that the dPEDE-A@M32 nano-vaccine induces immune memory, reshapes the tumor immune microenvironment, and suppresses tumor recurrence.

### dPEDE-A@M32 Nano-vaccine Elicits Systemic Immunity to Effectively Inhibit Hematogenous Metastasis of Cancer Cells

**Figure 6:**
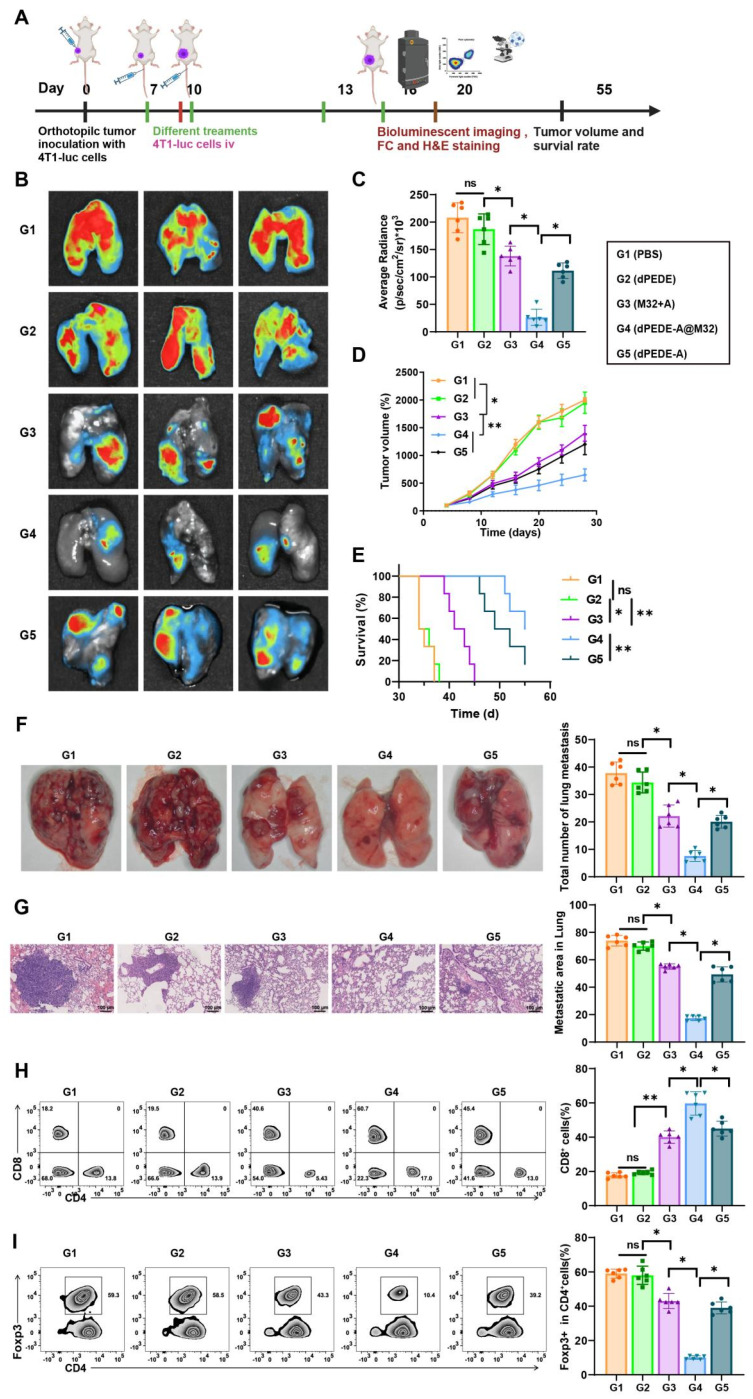
** Observing the therapeutic efficacy of nano-vaccine on hematogenous metastasis of BC.** Note: (A) Schematic of the hematogenous metastasis model, in which 4T1-luc breast cancer cells were intravenously injected into tumor-bearing mice on day 10, followed by treatment with PBS, dPEDE, M32+A, dPEDE-A, and dPEDE-A@M32 at specific time points; (B-C) *In vitro* bioluminescence imaging (BLI) of the lungs on day 20 post-treatment, with quantification (C). Representative images of three mice per group are shown (B) and corresponding statistical analysis (C), displaying three representative mice per group; (D) Tumor growth curves of the hematogenous metastasis model treated with PBS, dPEDE, M32+A, dPEDE-A, and dPEDE-A@M32; (E) Survival curves of the hematogenous metastasis model treated with PBS, dPEDE, M32+A, dPEDE-A, and dPEDE-A@M32; (F) Lung metastasis images and quantification of lung metastases in the PBS, dPEDE, M32+A, dPEDE-A, and dPEDE-A@M32 groups; (G) H&E staining of lung metastatic areas, with quantification of lung metastasis ratios, scale bar = 100 μm; (H) FCM analysis images and relative quantitative statistics of CD8^+^ T cells in the blood; (I) FCM analysis images and relative quantitative statistics of CD4^+^Foxp3^+^ Tregs in the blood. Each group in animal experiments consisted of six mice, and values are presented as mean ± SD. 'ns' indicates no significant difference between groups, * *p* < 0.05, ** *p* <0.01, *** *p* < 0.001.

Circulating cancer cells can invade various organs, with the lungs being the most common site of distant metastasis in BC, which leads to widespread cancer dissemination. We questioned whether our nano-vaccine could prevent hematogenous metastasis of BC 78-80. In a murine model of BC, we simulated tumor invasion and blood-borne metastasis by intravenously injecting 4T1-luc cancer cells (Figure 6A). Compared to spontaneous lung metastasis, the systemic metastasis model exhibited greater invasiveness and posed a significant challenge, making it suitable for evaluating specific metastasis inhibition 81, 82.

We monitored tumor growth and survival curves and collected lung specimens for *in vitro* analysis using bioluminescence imaging. As shown in Figure 6B-E, mice treated with PBS and dPEDE displayed severe lung metastatic lesions, while those treated with M32+A and dPEDE-A showed a slight reduction in bioluminescence intensity and primary tumor growth, indicating partial improvement in metastasis inhibition. Compared to the M32+A and dPEDE-A treatment group, the dPEDE-A@M32 group exhibited marked clearance of lung metastatic signals and substantial inhibition of the primary tumor. Lung imaging revealed almost no visible signs of metastasis in the dPEDE-A@M32 group, indicating effective suppression of cancer cell spread to the lungs, with significant differences in lung metastasis severity among groups (Figure 6F). H&E staining assessed lung invasion by cancer cells, with dashed outlines indicating metastatic nodules and their proportional area calculated. The dPEDE-A@M32 group showed a significant reduction in the number of metastatic nodules compared to the M32+A and dPEDE-A group (Figure 6G).

To elucidate the anti-metastatic mechanisms of dPEDE-A@M32, we analyzed the systemic anti-tumor immune responses following various treatments. FCM analysis revealed that dPEDE-A@M32 significantly enhanced CD8^+^ CTL activity and reduced Tregs (Figure 6H-I). The dPEDE-A@M32 nano-vaccine triggered T-cell-mediated cancer cell elimination in the bloodstream, thereby inhibiting systemic progression.

These findings demonstrate that the dPEDE-A@M32 nano-vaccine effectively stimulates systemic immunity, suppressing hematogenous metastasis of cancer cells.

### dPEDE-A@M32 Combined with Anti-PD-1 Enhances Therapeutic Efficacy Against BC

**Figure 7:**
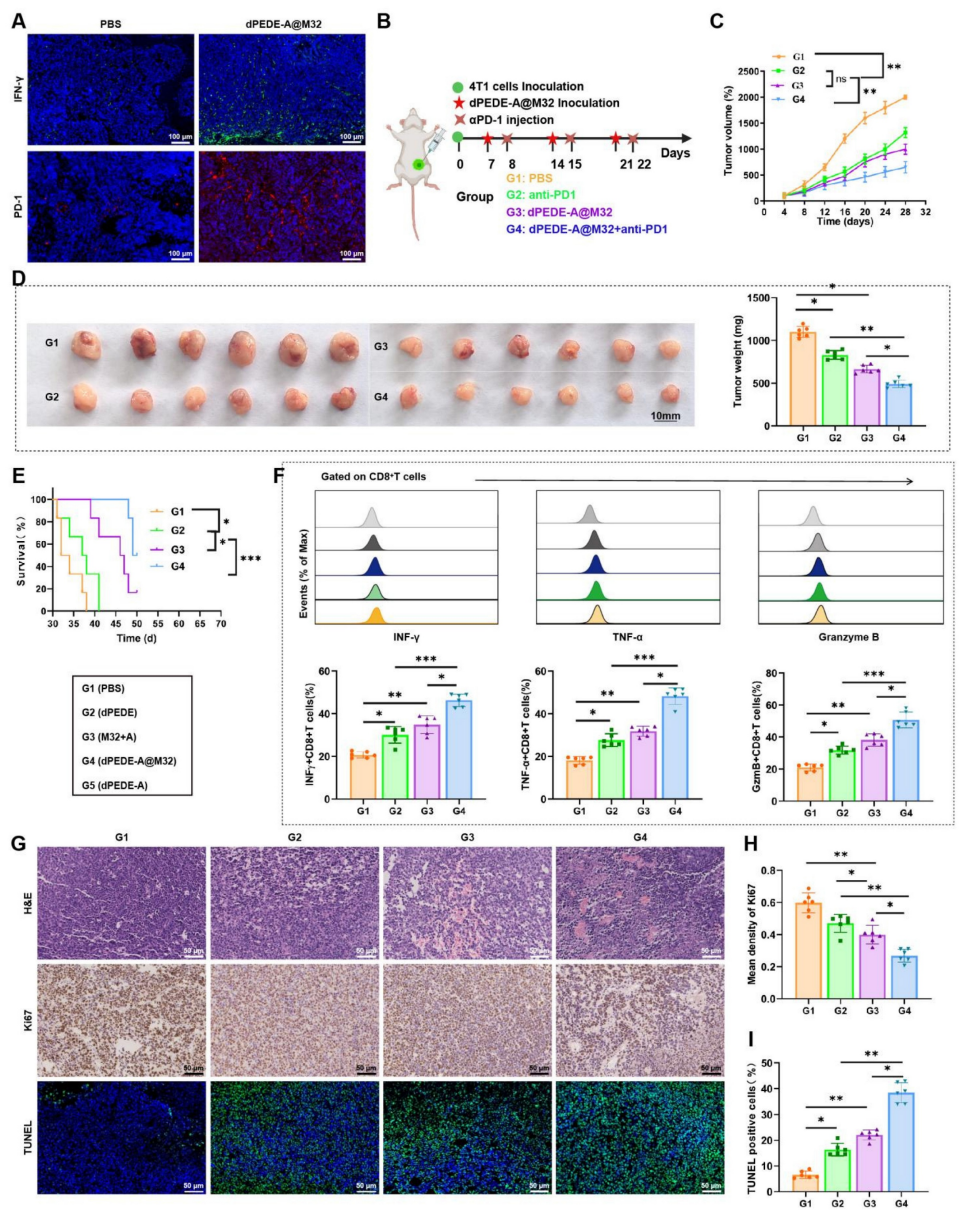
** Impact of nano-vaccine combined with ICB therapy on BC mice.** Note: (A) Immunostaining images of tumor tissues for IFN-γ and PD-1 post-nano-vaccine treatment, bar = 100μm; (B) Schematic diagram of the combined treatment process; (C) Tumor growth curves for the BC mouse groups; (D) Display of tumor volume and mass on day 28 post-treatment in each BC mouse group; (E) Survival curves for the BC mouse groups; (F) Flow cytometer analysis showing the proportion of INFγ^+^CD8^+^ T cells, TNF-α^+^CD8^+^ T cells, and GzmB^+^CD8^+^ T cells in tumor tissues of BC mice across different groups; (G-I) Tumor sections stained with H&E, Ki67, and TUNEL (G), statistical graph of Ki67 positive cells (H), and statistical graph of TUNEL positive cells (I), bar = 50 μm. Each group in animal experiments consisted of six mice, and values are presented as mean ± SD. 'ns' indicates no significant difference between groups, * *p* < 0.05, ** p <0.01, *** *p* < 0.001.

In previous experiments using a hematogenous metastasis model, preliminary immunohistochemical staining of tumor tissues showed significant increases in IFN-γ and PD-1 expression within the dPEDE-A@M32 treatment group (Figure 7A). To further explore its therapeutic potential, we investigated the efficacy of combining dPEDE-A@M32 with anti-PD-1 in treating BC. We established a BC model in BALB/c mice via subcutaneous injection of 4T1 cells and applied a combined treatment of dPEDE-A@M32 and anti-PD-1 to evaluate potential therapeutic enhancements (Figure 7B). Results indicated that both dPEDE-A@M32 and anti-PD-1 alone slowed tumor growth in mice; however, their combination exhibited a significantly greater inhibitory effect, leading to a marked reduction in tumor volume (Figure 7C-D). Survival analysis further confirmed that the combination therapy outperformed single-agent treatment (Figure 7E).

Subsequent FCM analysis of the tumor-infiltrating CD8^+^ T cells in the mice revealed increased expression of INFγ, TNF-α, and GzmB across all treatments, with the combined treatment showing the most pronounced effects (Figure 7F). Additionally, to assess the impact of single and combined treatments on tumor proliferation, apoptosis, and necrosis, H&E, Ki67, and TUNEL staining analyses were performed. The combination treatment group exhibited the highest apoptosis rates and the lowest proliferation rates. While single-agent treatments effectively inhibited tumor growth, the combined dPEDE-A@M32 and anti-PD-1 therapy significantly enhanced therapeutic outcomes in BC mice (Figure 7G-I).

Collectively, these findings indicate that combining dPEDE-A@M32 with anti-PD-1 enhances therapeutic efficacy in BC mice, highlighting its potential for clinical application.

## Discussion

BC remains one of the most common cancers among women globally, posing a significant threat to women's health 83-85. With lifestyle changes and an aging population, BC incidence continues to increase 86-88. Although current treatments, including surgery, radiotherapy, chemotherapy, and endocrine therapy, have shown success in early-stage BC, their effectiveness diminishes in advanced or recurrent cases, often leading to recurrence and metastasis 51, 89, 90. In recent years, immunotherapy, particularly ICIs, has provided new hope for BC treatment. However, significant challenges persist, especially in achieving effective immune activation to recognize and eliminate tumor cells 24. Therefore, developing innovative therapeutic approaches to enhance treatment outcomes and improve BC patients' quality of life remains a key research focus 91-93. This study explores a novel combination immunotherapy approach that integrates nanotechnology with ICIs, aiming to overcome current treatment limitations and provide more effective solutions for BC therapy.

Nanotechnology is increasingly central to cancer treatment, particularly in the development of tumor-specific drug delivery systems 94-96. Compared to traditional therapies, nano-vaccines offer greater precision and lower toxicity to normal tissues 97-99. The pH-responsive nano-vaccine developed in this study utilizes advanced RAFT polymerization technology, allowing for more effective drug release in the tumor's mildly acidic environment—a capability rarely explored in previous studies. By chemically conjugating the STING agonist ADU-S100 with neoantigens, this study not only enhances nano-vaccine stability but also strengthens immune activation. This design significantly enhances the vaccine's targeting and immunomodulatory effects, providing new avenues for treating solid tumors such as BC.

Although anti-PD-1 antibodies have shown significant effects in the treatment of various cancers, their efficacy in BC remains limited, primarily due to the complexity of the BC immune microenvironment and the heterogeneous PD-L1 expression by tumor cells 51, 100, 101. This study explores the combined use of a nano-vaccine with anti-PD-1 antibodies, aiming to enhance immune responses through two mechanisms: the nano-vaccine activates DCs and specific T-cells, while the anti-PD-1 antibody blocks the immune checkpoint, relieving immune suppression. This dual strategy has demonstrated greater tumor suppression than individual treatments, outperforming previous approaches that relied on single-agent therapies.

Modulating the tumor microenvironment is critical for improving therapeutic outcomes in cancer treatment 102-104. In this study, the nano-vaccine transforms the immunosuppressive state of the tumor microenvironment by activating DCs and promoting immune cell migration and infiltration. This activation is not limited to a single type of immune cell but encompasses a broad range of immune cells, including macrophages and natural killer cells, thereby creating a comprehensive immune response. Unlike previous research focused on a single immune cell activation, this study provides a strategy for broad immune modulation within the tumor microenvironment. Moreover, the nano-vaccine's design enables precise drug release, ensuring activation in the tumor's mildly acidic environment—a challenge often unmet in previous studies—highlighting its potential for precision medicine. This targeted release not only enhances therapeutic efficacy but also significantly reduces systemic side effects, reinforcing the role of nanotechnology in improving both the safety and effectiveness of cancer treatments. To reduce systemic toxicity, we optimized the formulation by minimizing the PEI dosage and incorporating PEG into the polymer structure. Additionally, this is not our first study utilizing PEI. Previous studies 37, 38, 39, 42, 43 have employed PEI-based polymers in cancer therapy, with no significant toxicity observed.

Effective immunotherapy should not only eliminate existing tumor cells but also prevent recurrence 105, 106. In this study, the nano-vaccine activates effector memory cells, offering long-term immune protection. This strategy is particularly crucial in BC treatment, where recurrence and metastasis are major causes of therapeutic failure. The nano-vaccine combined with anti-PD-1 therapy not only enhanced the primary immune response but also expanded and sustained the memory T cell population, strengthening the immune system's capacity for long-term tumor surveillance. Compared to previous studies, this research demonstrates that combining nanotechnology with ICIs can more effectively activate immune memory, providing more durable anti-tumor protection. This prolonged immune memory could potentially lower recurrence rates in future therapies, improving overall patient prognosis, and offering new strategies for immunotherapy in BC and other tumor types.

The main innovation of this study lies in the development of a novel pH-sensitive nano-vaccine that integrates a STING agonist and a breast cancer neoantigen (M32) into a polymeric system, specifically designed for synergistic use with anti-PD-1 therapy. This pH-sensitive nano-vaccine incorporates pH-responsive polymers to efficiently co-deliver antigens and adjuvants, optimizing controlled release and co-delivery in the acidic tumor microenvironment. The nano-vaccine enhanced immune memory, demonstrating greater efficacy in preventing tumor metastasis and recurrence compared to conventional strategies. Furthermore, this nano-vaccine exhibited synergistic therapeutic potential with ICIs (anti-PD-1) in breast cancer models, showing significant combinatory effects. Although this study has yielded important theoretical and experimental insights, it has limitations. First, while the mouse model used simulates the human BC immunotherapeutic response, physiological and immunological differences may affect the applicability of the experimental results to humans. Second, although the nano-vaccine demonstrated good biocompatibility and low toxicity, the long-term safety and potential side effects still require evaluation in larger-scale clinical trials. Moreover, detailed pharmacokinetic studies are planned to further optimize its clinical potential. Finally, while this study focused on the activation and persistence of immune memory, further investigation is needed into its effects and mechanisms across different BC subtypes, particularly those with low immunogenicity.

Looking ahead, research in this field will focus on several key issues. First, future studies should employ more complex biological models, such as humanized mouse models or large animals with intact immune functions, to better predict the clinical outcomes of nano-vaccine and ICI combination therapies. Second, there will be efforts to further optimize nano-vaccine design, including targeting additional immunoregulatory molecules or developing smart nano-vaccines capable of responding to multiple biomarkers, thereby enhancing treatment specificity and minimizing side effects. Additionally, exploring the combination of nano-vaccines with other therapeutic modalities such as radiotherapy, chemotherapy, and targeted therapies could provide more effective strategies for treating complex and refractory BC. Finally, a deeper investigation into the mechanisms of treatment, particularly how immune cells interact within the tumor microenvironment, will aid in developing new immunoregulatory therapeutic strategies, ultimately improving survival rates and quality of life for BC patients.

## Conclusion

This study successfully developed a targeted pH-responsive nano-vaccine that encapsulates a STING agonist and neoantigens, significantly enhancing the immunotherapeutic effects against BC. The nano-vaccine was validated in co-culture experiments with murine DCs, demonstrating its ability to activate the immune system and enhance antigen presentation. Moreover, the combination of the nano-vaccine with anti-PD-1 therapy exhibited outstanding efficacy in inhibiting tumor growth and metastasis in an *in vivo* mouse model, particularly in preventing tumor recurrence and promoting long-term survival.

These findings underscore the potential of targeted nano-vaccines in activating specific immune responses and enhancing T-cell-mediated tumor clearance. Furthermore, combining anti-PD-1 therapy with a nano-vaccine effectively overcomes the limitations of single ICI treatments, offering a promising approach to enhance immune responses and improve BC prognosis. These results are particularly significant for research seeking more effective clinical strategies for treating BC, especially for advanced or treatment-resistant BC.

Despite the promising outcomes, there are several limitations. First, the research was primarily conducted in mouse models, which may differ from human physiology and immune responses. Therefore, further validation in a broader range of animal models and even clinical trials is necessary. Second, the long-term safety and potential immune-related side effects of the nano-vaccine have not been fully assessed, necessitating deeper investigation in future studies. Additionally, the complex and costly preparation process may hinder its application in low-resource settings.

Despite these limitations, this study provides valuable insights and new strategies for the immunotherapy of BC. Future research should focus on optimizing the design and production process of the nano-vaccine to ensure its efficacy and safety in clinical applications, and to explore its broader applications in other cancers.

## Supplementary Material

Supplementary figures and tables.
