# Supplementary material for: pH-responsive nano-vaccine combined with anti-PD-1 antibodies for enhanced immunotherapy of breast cancer
Source: Theranostics. 2025 Apr 28;15(12):6022–43. doi: 10.7150/thno.107200 (PMC12068292; doi:10.7150/thno.107200)
Supplement: Supplementary file 1 — Supplementary figures and tables. [file thnov15p6022s1.pdf]

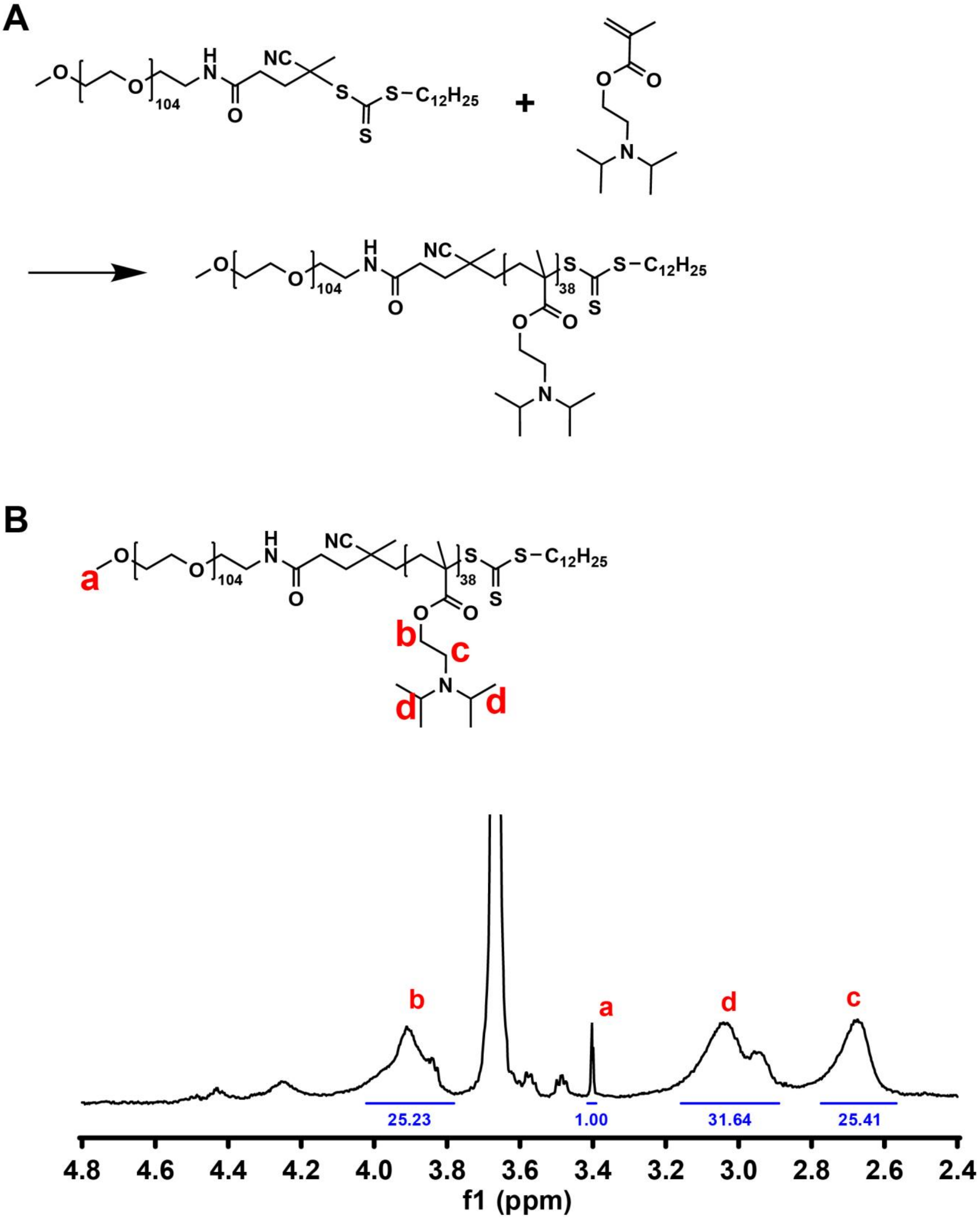

A
104
S−C12H25
+
104
38
S−C12H25
B
a
104
38
S−C12H25
b
c
d
d
b
a
d
c
25.23
1.00
31.64
25.41
4.8
4.6
4.4
4.2
4.0
3.8
3.6
3.4
3.2
3.0
2.8
2.6
2.4
f1 (ppm)

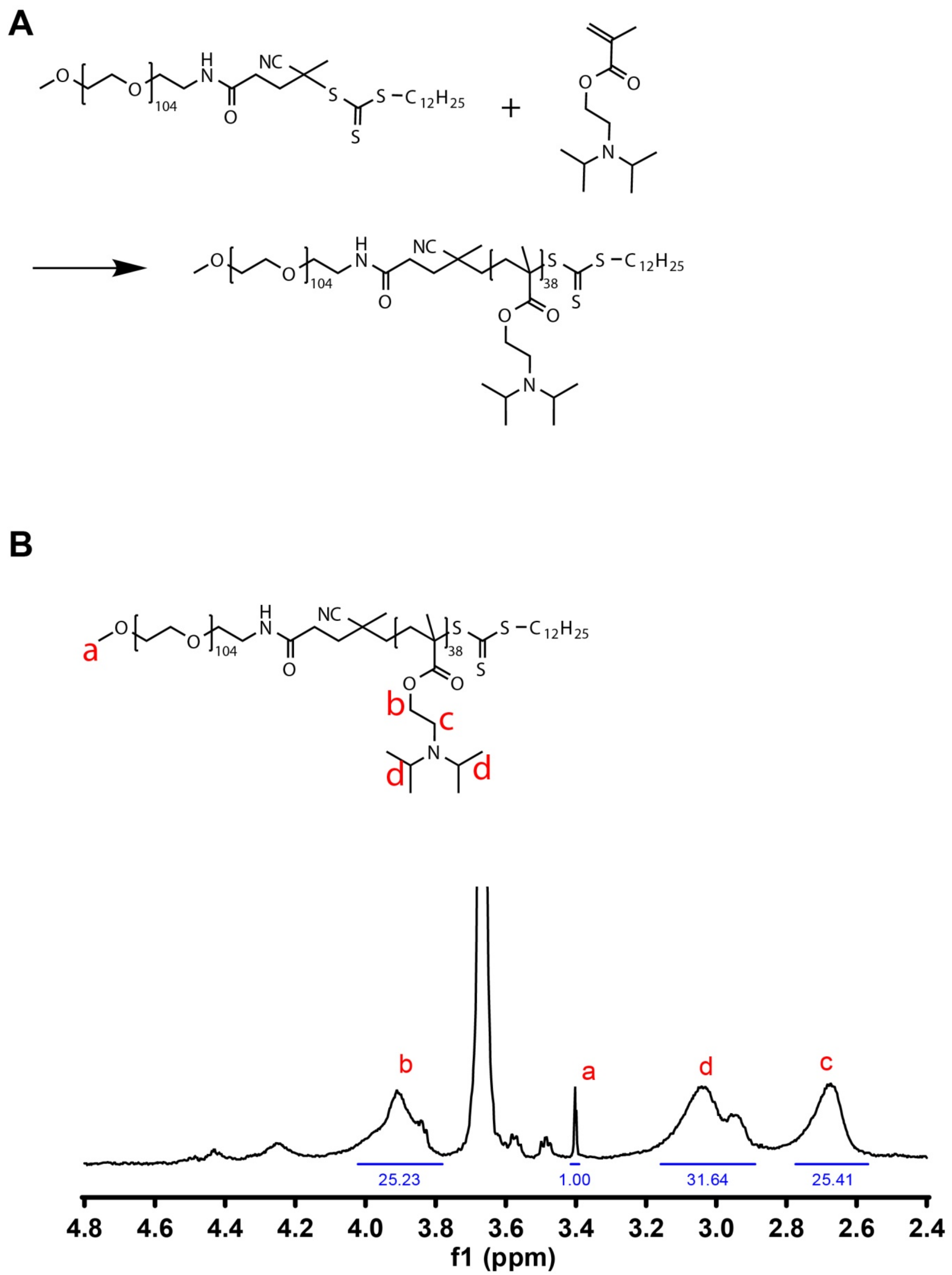

Figure S1. Characterization of PEG_{108}-b-$PDPA_{38}$ Diblock Copolymer

Note: (A) Schematic illustration of the molecular structure for the synthesis of PEG_{108}-b-$PDPA_{38}$ via RAFT polymerization; (B) ^{1}H-NMR spectrum of PEG_{108}-b-$PDPA_{38}$ diblock copolymer.

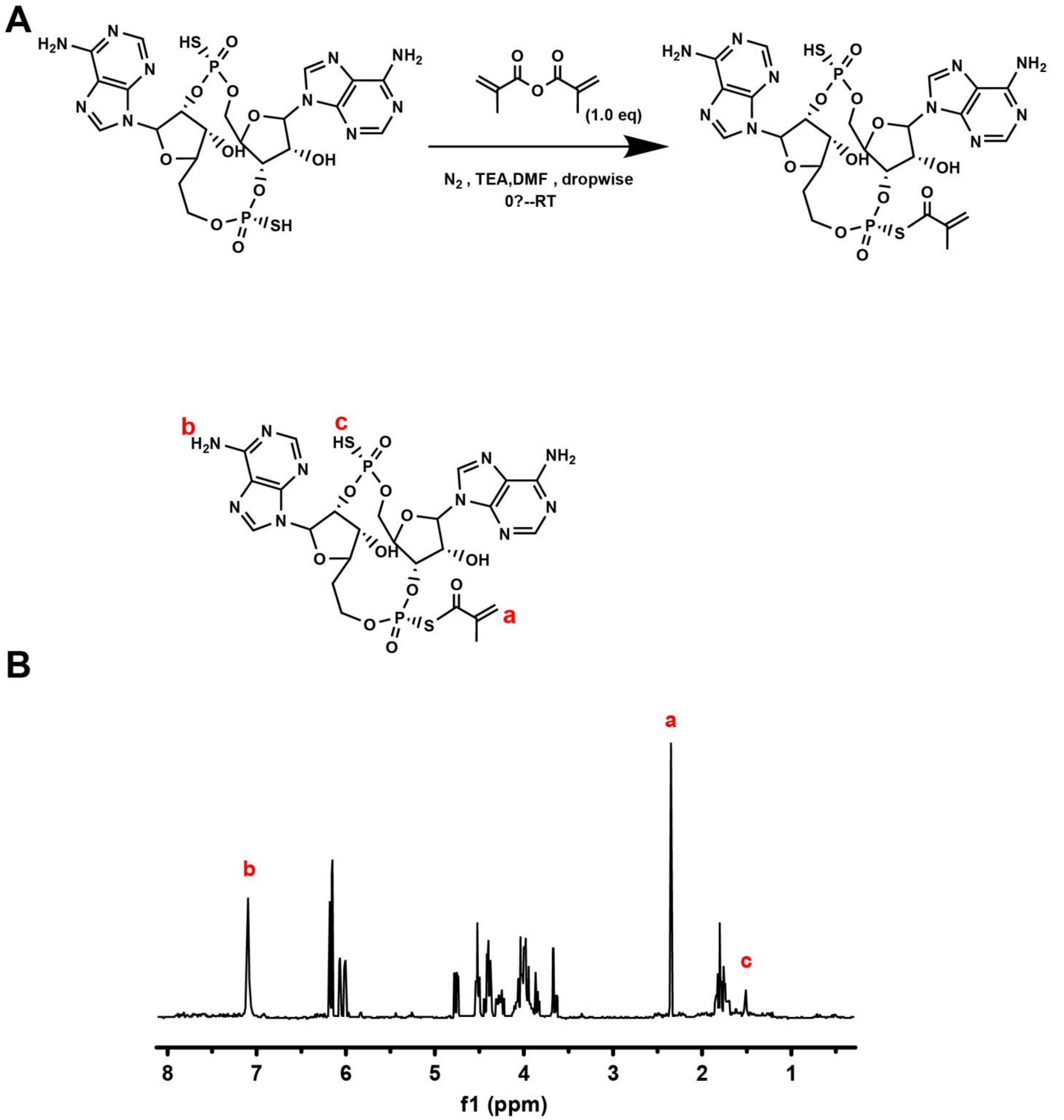

Figure S2. Characterization of ADU-S100 Methacrylate (ADU-S100-MA)

Note: (A) Schematic illustration of ADU-S100 methacrylate modification; (B) ^{1}H-NMR spectrum of ADU-S100-MA

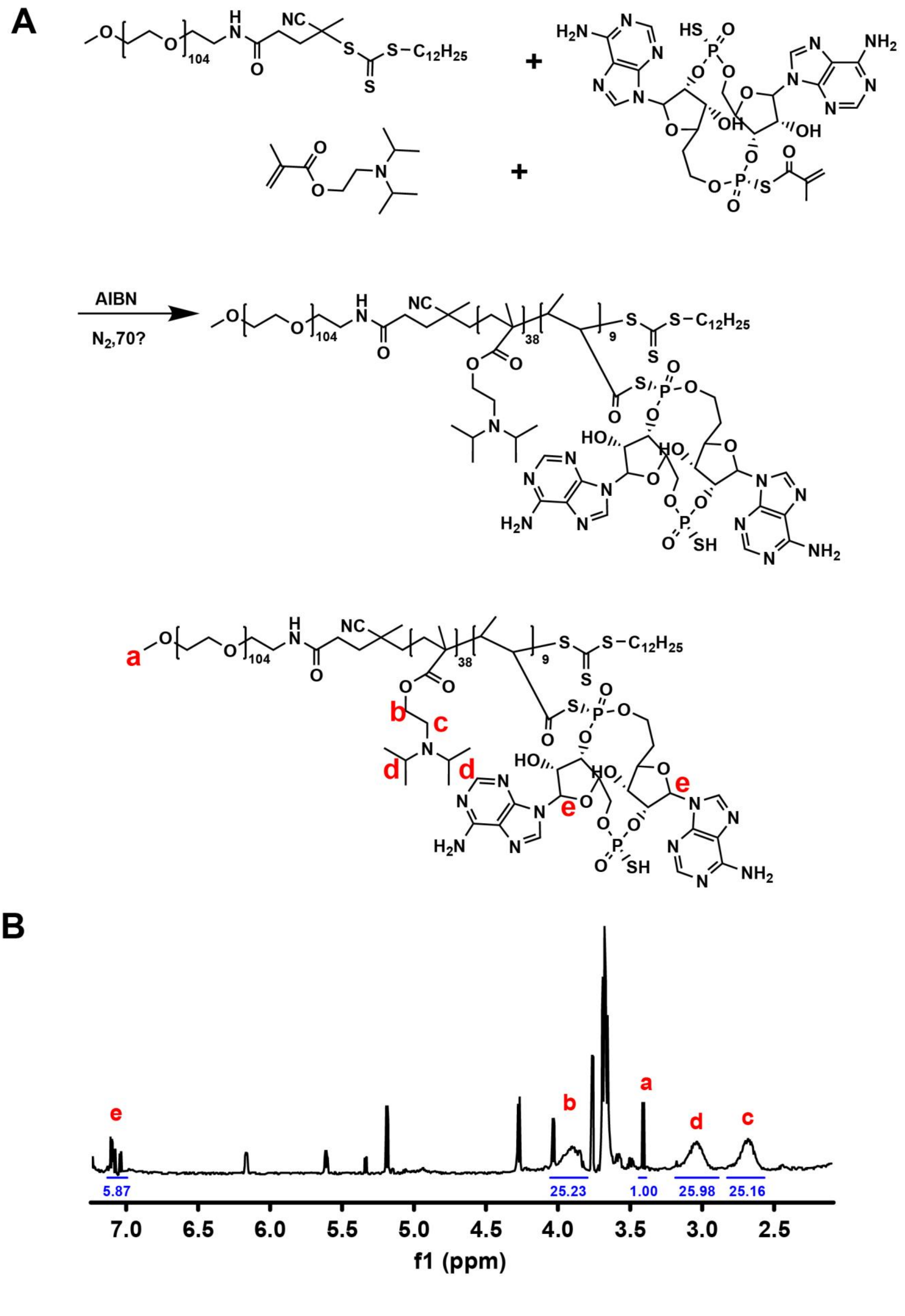

Figure S3. Characterization of PEG-b-P(DPA-ADU-S100)

Note: (A) Schematic illustration of the molecular structure of PEG-b-P(DPA-ADU-S100); (B)

^{1}H-NMR spectrum of PEG-b-P(DPA-ADU-S100).

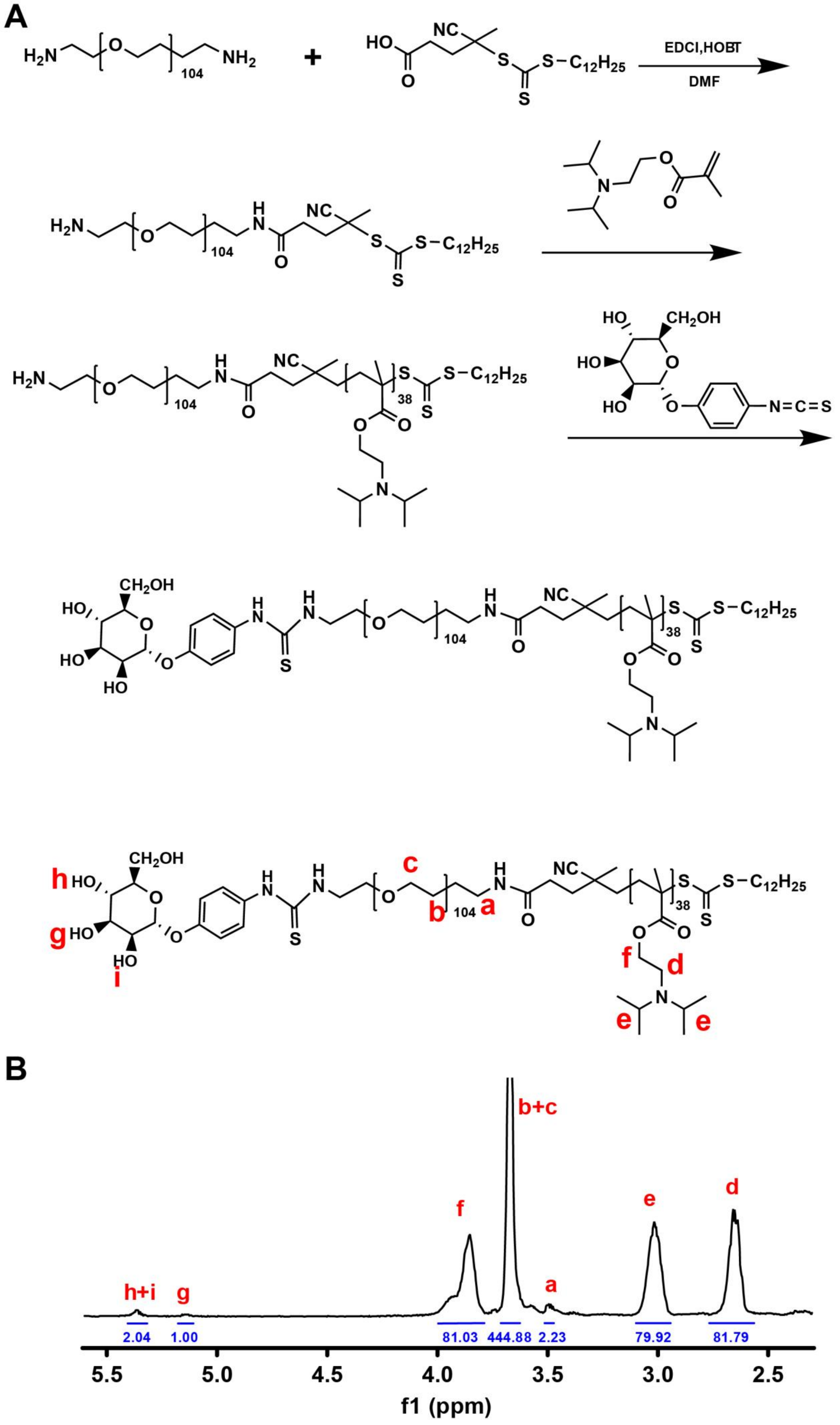
A
EDCI,HOBT
DMF
104
38
$C_{12}H_{25}$
CH_2OH
N=C=S
a
b
c
d
e
f
g
h
i
B
h+i
g
f
b+c
a
e
d
2.04
1.00
81.03
444.88
2.23
79.92
81.79
5.5
5.0
4.5
4.0
3.5
3.0
2.5
f1 (ppm)

Figure S4. Characterization of Dex-PEG-b-P(DPA)

Note: (A) Schematic illustration of the molecular structure of Dex-PEG-b-PDPA; (B)^{1}H-NMR spectrum of Dex-PEG-b-PDPA.

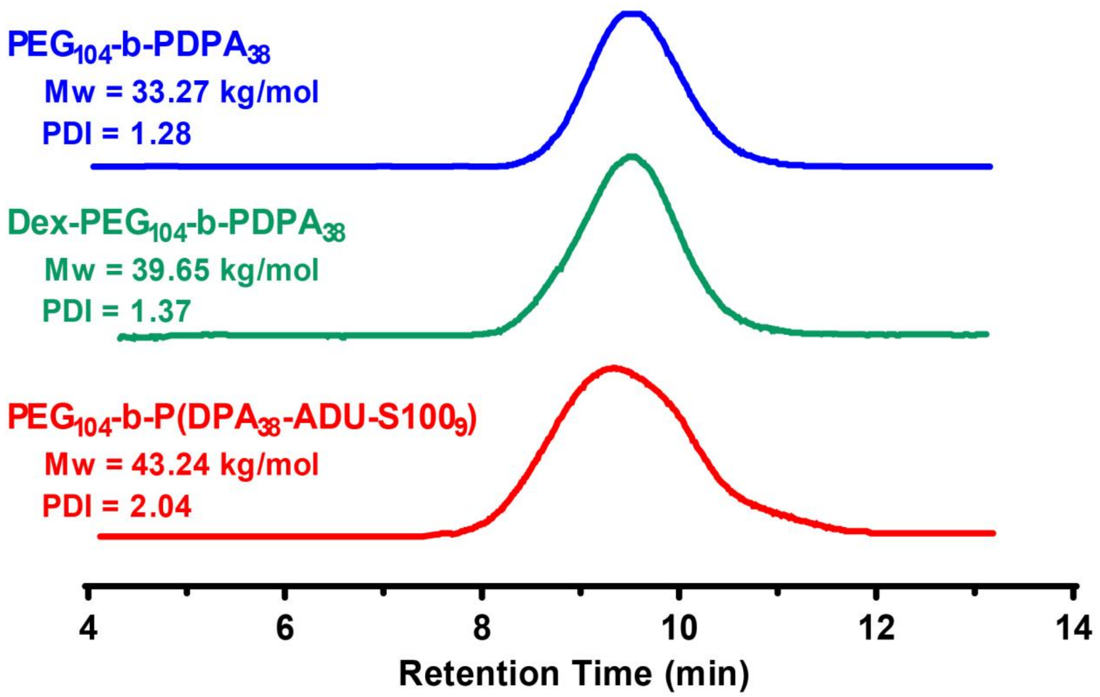

Figure S5. GPC Analysis of Key Polymers

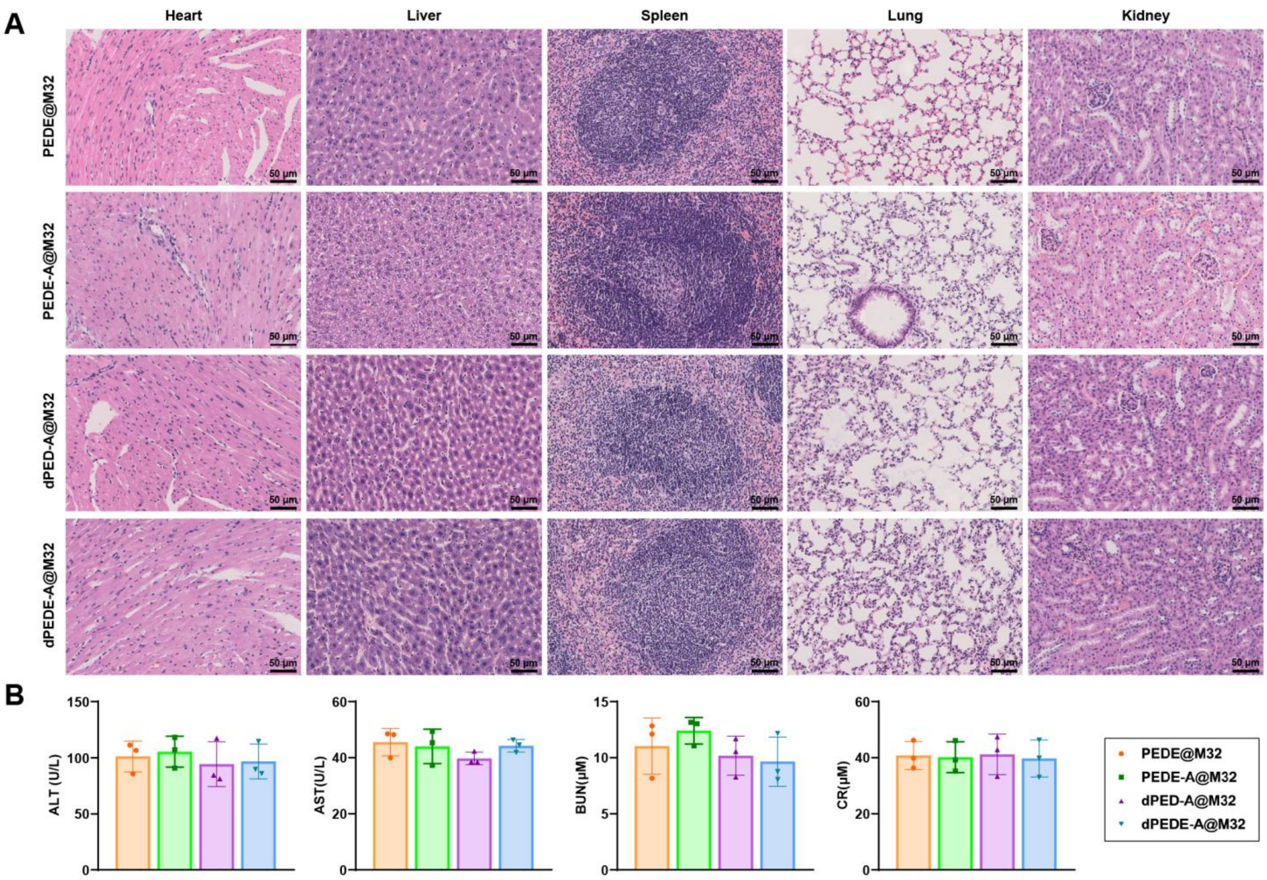

Figure S6. Distribution of nano-vaccine *in vivo*.

Note: (A) Representative fluorescence images of major organs (from left to right: heart, liver, spleen, lungs, and kidneys) 7 days post-administration; (B) Biodistribution analysis of nano-vaccines in major organs and LNs at day 7 after administration of PEDE@M32, PEDE-A@M32, dPED-A@M32, and dPEDE-A@M32. Each group in the animal experiments consisted of three mice, and values are presented as mean ± SD. 'ns' indicates no significant difference between groups, * $p < 0.05$, ** $p < 0.01$, *** $p < 0.001$.

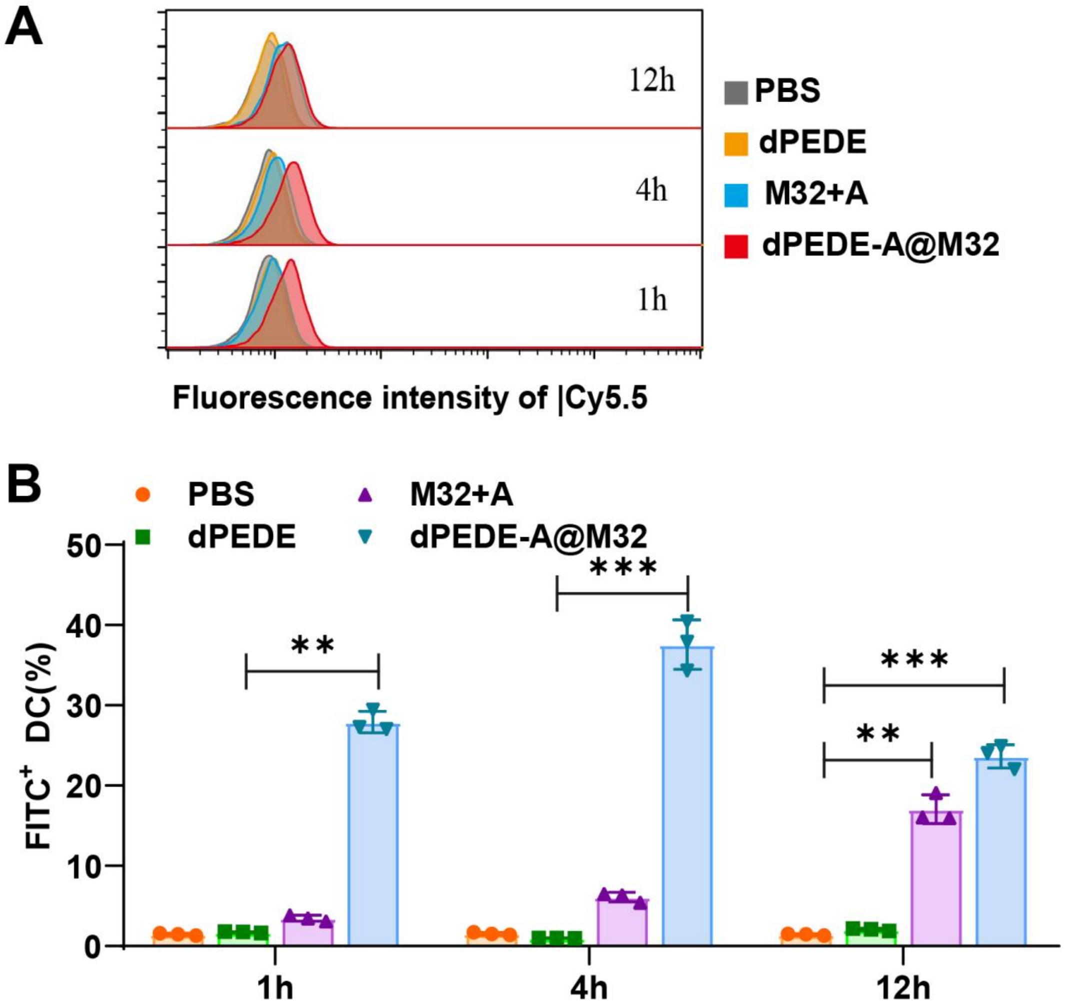

Figure S7. Biocompatibility assessment of the nano-vaccine.

Note: (A) H&E staining of heart, liver, spleen, lung, and kidney tissues from mice in different groups to observe pathological changes, bar = 50μm; (B) Biodistribution analysis of nano-vaccines in major organs and LNs at day 7 after administration of PEDE@M32, PEDE-A@M32, dPED-A@M32, and dPEDE-A@M32.

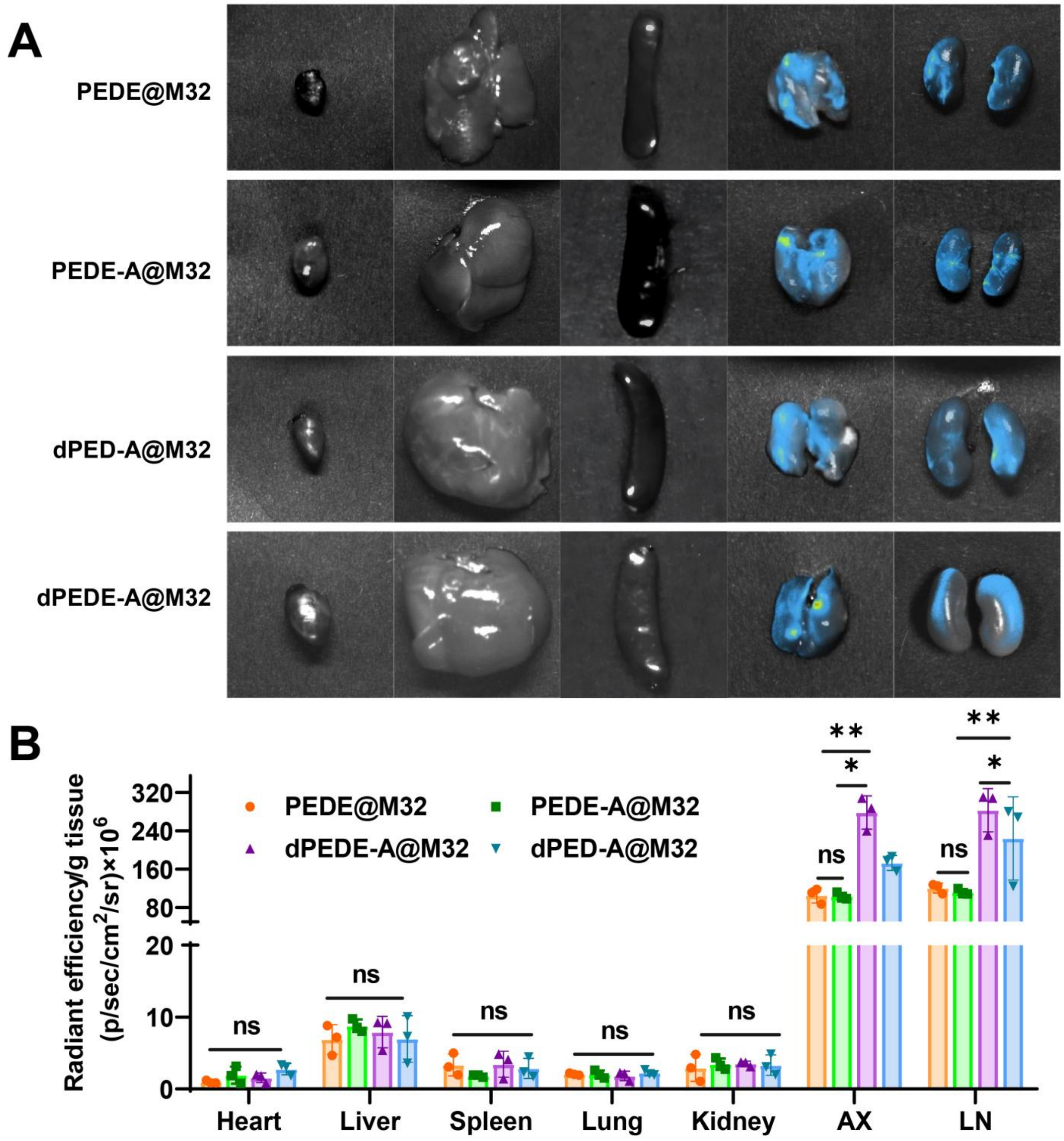

Figure S8. FCM analysis of DC cell uptake of nanomaterials.

Note: BMDCs were co-incubated with free M32-FITC+ADU-S100, blank carrier dPEDE, and nano-vaccine dPEDE-A@M32-FITC, which were designated as M32+A, dPEDE, and dPEDE-A@M32, respectively. FCM analysis was performed at 1, 4, and 12 hours post-incubation to evaluate uptake efficiency. (A) and (B) statistical chart. The experiment was repeated three times. Values are presented as mean ± SD. * indicates a significant difference ($p < 0.05$) between M32+A group and PBS group, while *** indicates a highly significant difference ($p < 0.001$) between

dPEDE-A@32 group and PBS group.

Table S1. Abbreviated noun explanation.

Abbreviation	Component
PEG	Polyethylene Glycol
DPA	diisopropylamino ethyl methacrylate
PED	PEG-*b*-PDPA,
PED-A	PEG-*b*-(PDPA-ADU-S100)
dPED	(Dex-PEG)-*b*-PDPA
PEDE	PEG-*b*-PDPA：PEI=3:1 micellar nanoparticles
PEDE-A	PEG-*b*-PDPA：PEG-*b*-(PDPA-ADU-S100)：PE1=1:2:1 micellar nanoparticles
dPEDE-A	(Dex-PEG)-*b*-PDPA：PEG-*b*-(PDPA-ADU-S100)：PE1=1:2:1micellar nanoparticles
dPEDE/A	dPEDE and ADU-S100 physical blending
dPEDE-A@M32	dPEDE-A nanomicelles loaded with M32 antigen peptide

Table S2. Primary antibody product details.

Name	Cat.	Species Reactivity	Ratio	Manufacturer	Country
P-STING	PA5-105674	Mouse	1:1000	Thermo Fisher	USA
STING	PA5-23381	Mouse	1:1000	Thermo Fisher	USA
P-TBK1	PA5-105919	Mouse	1:1000	Thermo Fisher	USA
TBK1	703154	Mouse	1:1000	Thermo Fisher	USA
P-IRF3	PA5-36775	Mouse	1:1000	Thermo Fisher	USA
IRF3	MA5-32348	Mouse	1:1000	Thermo Fisher	USA
β-actin	MA1-140	Mouse	1:1000	Thermo Fisher	USA
